# HPV16E1 downregulation altered the cell characteristics involved in cervical cancer development

**DOI:** 10.1038/s41598-023-45339-1

**Published:** 2023-10-25

**Authors:** Thanayod Sasivimolrattana, Arkom Chaiwongkot, Parvapan Bhattarakosol

**Affiliations:** 1 Medical Microbiology Interdisciplinary Program, Graduate School, Chulalongkorn University, Bangkok, 10330 Thailand; 2 Center of Excellence in Applied Medical Virology, Department of Microbiology, Faculty of Medicine, Chulalongkorn University, Bangkok, 10330 Thailand; 3 Division of Virology, Department of Microbiology, Faculty of Medicine, Chulalongkorn University, Bangkok, 10330 Thailand

**Keywords:** Tumour virus infections, Human papilloma virus, Virus-host interactions

## Abstract

The primary causes of cervical cancer are human papillomavirus type 16 (HPV16) and/or other high-risk (Hr −) HPV infections. Hr-HPVE5, E6, and E7 have been identified as oncoproteins that play roles in the development of cancer. However, other HPV proteins, especially E1, may also be involved in cancer development. In this study, the role of HPV16E1 in cervical carcinogenesis was examined by siRNA knockdown experiments using SiHa cells as a model. The results showed that HPV16E1 regulated P-FOXO3a and HPV16E7 expression. Various cell functions associated with the hallmarks of cancer, including cell viability, colony formation, invasion, and anchorage-independent cell growth, were altered when HPV16E1 was downregulated. However, no effect on cell migration and apoptosis properties was found. Moreover, HPV16E1 downregulation resulted in an increase in cisplatin susceptibility. In conclusion, this is the first demonstration that HPV16E1 might be regarded as a possible novel oncoprotein involved in several processes related to oncogenesis.

## Introduction

HPV is a circular, double-stranded DNA virus. The genetic material of this virus is contained in the naked icosahedral capsid and packaged as a minichromosome with cellular nucleosomal histone^1^. HPV belongs to the family *Papillomaviridae*, currently divided into 16 genera, of which 5 genera contain members that infect humans, i.e., *Alpha-, Beta-, Gamma-, Mu-,* and *Nupapillomavirus*^1^. Over 170 distinct types of HPV have been identified so far^2^. HPV is divided into two main categories based on the likelihood that cervical cancer will develop: low-risk (Lr-) HPVs and high-risk (Hr-) HPVs. There are at least 15 Hr-HPV types that have been identified, including types 16, 18, 31, 33, 35, 39, 45, 51, 52, 56, 58, 59, 68, 73, and 82^2^. Among Hr-HPV types, HPV16 is the most prevalent, and is frequently found in more than 50% of cervical carcinoma cases^3,4^. In addition, Hr-HPV infection has been linked to cancer in other body parts, including head and neck squamous cell carcinomas (HNSCC)^5,6^, oral cancer^7^, and penile cancer^8^.

Six early proteins—E1, E2, E4, E5, E6, and E7—and two late proteins—L1 and L2—are encoded by the HPV genome^9^. Early proteins take part in viral DNA replication by interacting with cellular gene products, whereas late proteins are responsible for the viral capsid's structural components^10^. There are diverse transcriptional patterns in HPV’s genes^11^. Some genes usually express from major monocistronic transcript, for example, E1, whereas many genes are polycistronic mRNAs, e.g., E6 and E7^12,13^. To this date, Hr-HPV E5, E6, and E7 proteins have been recognized as significant oncoproteins^14–16^. However, there is some evidence that other HPV proteins contribute to oncogenesis, such as E1, the main replicative helicase protein^17^. Recently, HPV16 E1 role in the emergence of cervical cancer has been investigated^18^. It was demonstrated that there is a positive correlation between E1 mRNA expression and cervical cancer progression (SCC > CIN2/3 > CIN1 > normal)^18^. In addition, the expression of the forkhead box O3 (FOXO3) protein, a protein that acts as an apoptosis regulator by upregulating genes necessary for cell death, was one of the genes that were mostly down-regulated in HPV16 E1 overexpressing HEK 293 T cells, according to the results of the microarray and real-time RT-PCR^19^. Therefore, FOXO3a downregulation in HPV16 E1-expressing cells may promote cell growth and survival. However, no study reveals the association between HPV16 E1 and the FOXO3 protein.

In the current study, we identified the potential function of HPV16E1 in cervical carcinogenesis through siRNA knockdown experiments. Functions related to carcinogenesis were observed, such as cell growth, proliferation, apoptosis, invasion/migration, and anchorage-independent cell growth. In addition, the association between HPV16E1 and FOXO3a was also demonstrated.

## Results

### Effect of HPV16E1 knocked down on HPV gene expression

**Figure 1:**
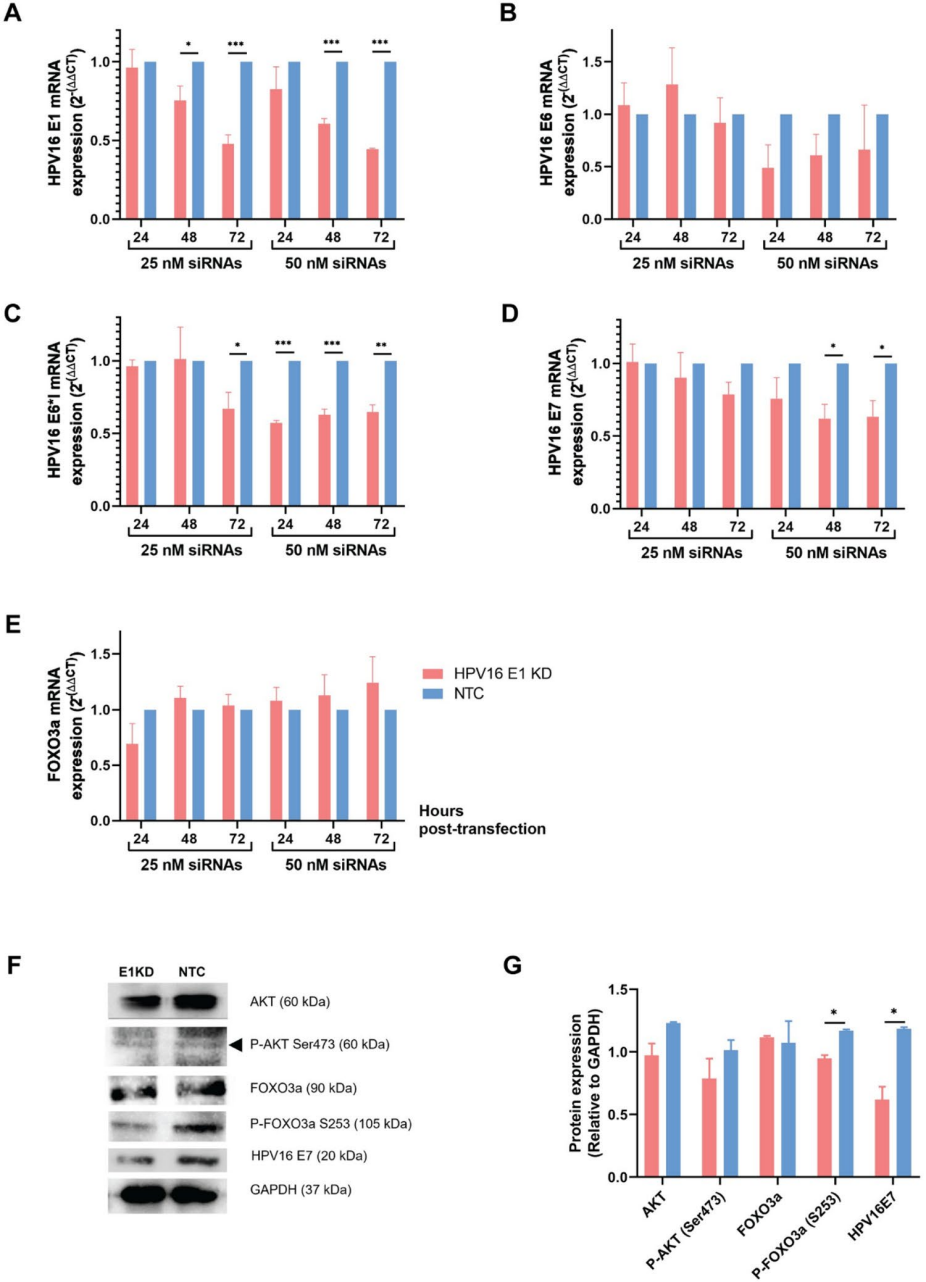
The expression of mRNA and protein expression in E1KD SiHa cells. (**A**–**E**) mRNA expression; (**A**) HPV16E1 (**B**) HPV16E6 (**C**) HPV16E6*I (**D**) HPV16E7 (**E**) FOXO3a. (**F**,**G**) AKT, P-AKT, FOXO3a, P-FOXO3a, and HPV16E7 protein expression; (**F**) Cropped protein bands detected by western bolt (**G**) Relative ratio between each protein and GAPDH. The blots were cut prior to hybridization with antibodies. The intensity of each protein band was quantified by image J software. Asterisks indicated a significant difference (*p < 0.05, ** *p* < 0.01, *** *p* < 0.001, *unpaired t-test*). Error bars represent SEM.

25 and 50 nM siRNAs targeting HPV16E1, siRE1.3–6, were transfected into SiHa cells for 24, 48, and 72 h. SiHa cells that had been transfected with non-targeted siRNA (NTC) were used as a control. The expression of HPV16E1 was quantified using RT-qPCR at the specified time. The findings showed that after 72 h of 50 nM siRNA transfection, the expression of HPV16E1 declined by about 50% (Fig. 1A). According to some HPV transcripts, which are normally expressed as polycistronic^20^, the expression of other HPV genes, i.e., HPV16E6, E6*I, and E7, was also observed. No effect on the expression of HPV16E6 mRNA was demonstrated (Fig. 1B). In contrast, HPV16E6*I and E7 mRNA expression was significantly decreased after E1KD at 50 nM, especially at 48 and 72 h (Fig. 1C and D). The downregulation of E6*I and E7 mRNA are not an effect of E1siRNA targeted to E6*I and E7 mRNA. It is a consequence of the loss of function of HPV16E1 protein after E1 mRNA is knocked down since it is known that HPVE1 plays an important role in regulation of HPVE6 and E7 expression^21^. In addition, either HPV16E6KD or HPV16E7KD cells were performed to observe the effect on HPVE1 expression. As predicted, no downregulation of HPV16E1 was demonstrated in any E6/E7KD conditions (Supplemental Fig. 1A). On the other hand, the mRNA expression of HPV16E6, E6*I, and E7 was suppressed in E7KD and E6 + 7KD but not in E6KD (Supplemental Fig. 1B–D).

### P-FOXO3a cellular protein expression in HPV16E1 knockdown cells

The expression of the host transcript, FOXO3a mRNA, was also observed. The findings showed that FOXO3a mRNA expression was not significantly different at any time point; however, there was a trend toward increased expression at 48 and 72 h in E1KD SiHa cells (50 nM) (Fig. 1E). In addition, the expression of FOXO3a mRNA was decreased in E7KD and E6 + E7KD SiHa cells but not in E6KD (Supplemental Fig. 1E). The protein expression in the FOXO signaling pathway, such as AKT, P-AKT (Ser473), FOXO3a, and P-FOXO3a (S253) was determined in E1KD SiHa cells by SDS-PAGE and western blot. The results indicated that among those proteins, only P-FOXO3a (S253) was statistically reduced (Fig. 1F, G and Supplemental Fig. 2). Suppression of HPV16E7 protein (Fig. 1G) was confirmed to be related to the previous mRNA expression (Fig. 1D). Moreover, the phosphorylation rates of AKT and FOXO3a were compared between E1KD and NTC SiHa cells, and no significant difference was found. However, an increased tendency of AKT phosphorylation rate was shown, whereas a decreased tendency of FOXO3a phosphorylation rate was indicated (Supplemental Fig. 3).

**Figure 2:**
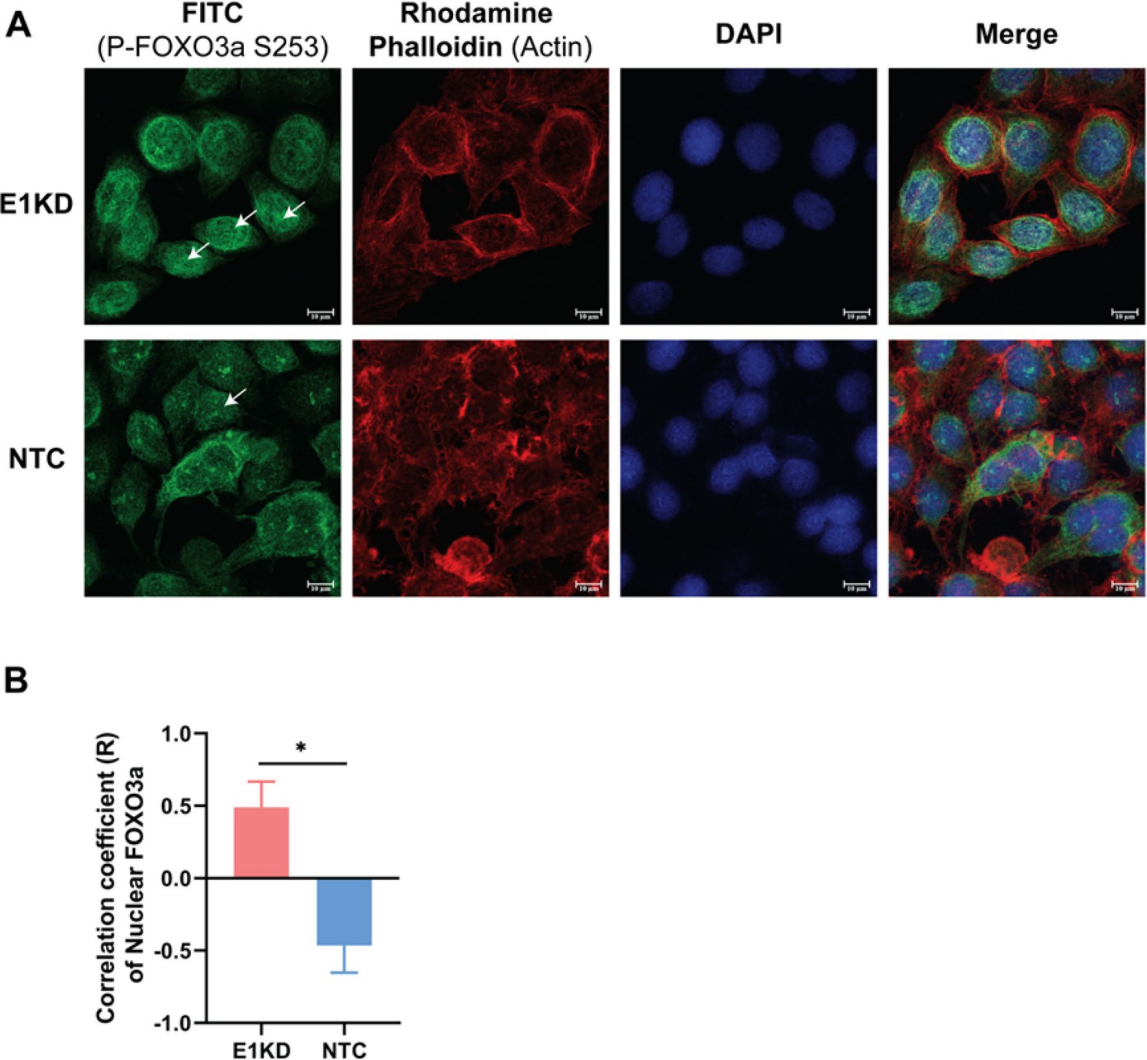
P-FOXO3a localization. The cells were stained by rabbit polyclonal antibody, rhodamine phalloidin, and DAPI for P-FOXO3a (green), actin (red), and nuclear (blue) staining, respectively. The cells were observed under a confocal microscope (6300x). The arrowhead indicated the nuclear P-FOXO3a. (**A**) In E1KD and NTC SiHa cells. (**B**) Correlation coefficient (R) of nuclear P-FOXO3a. Asterisks indicate a significant difference (*p < 0.05, *unpaired t-test*). Three independent experiments were conducted. Error bars represent SEM.

**Figure 3:**
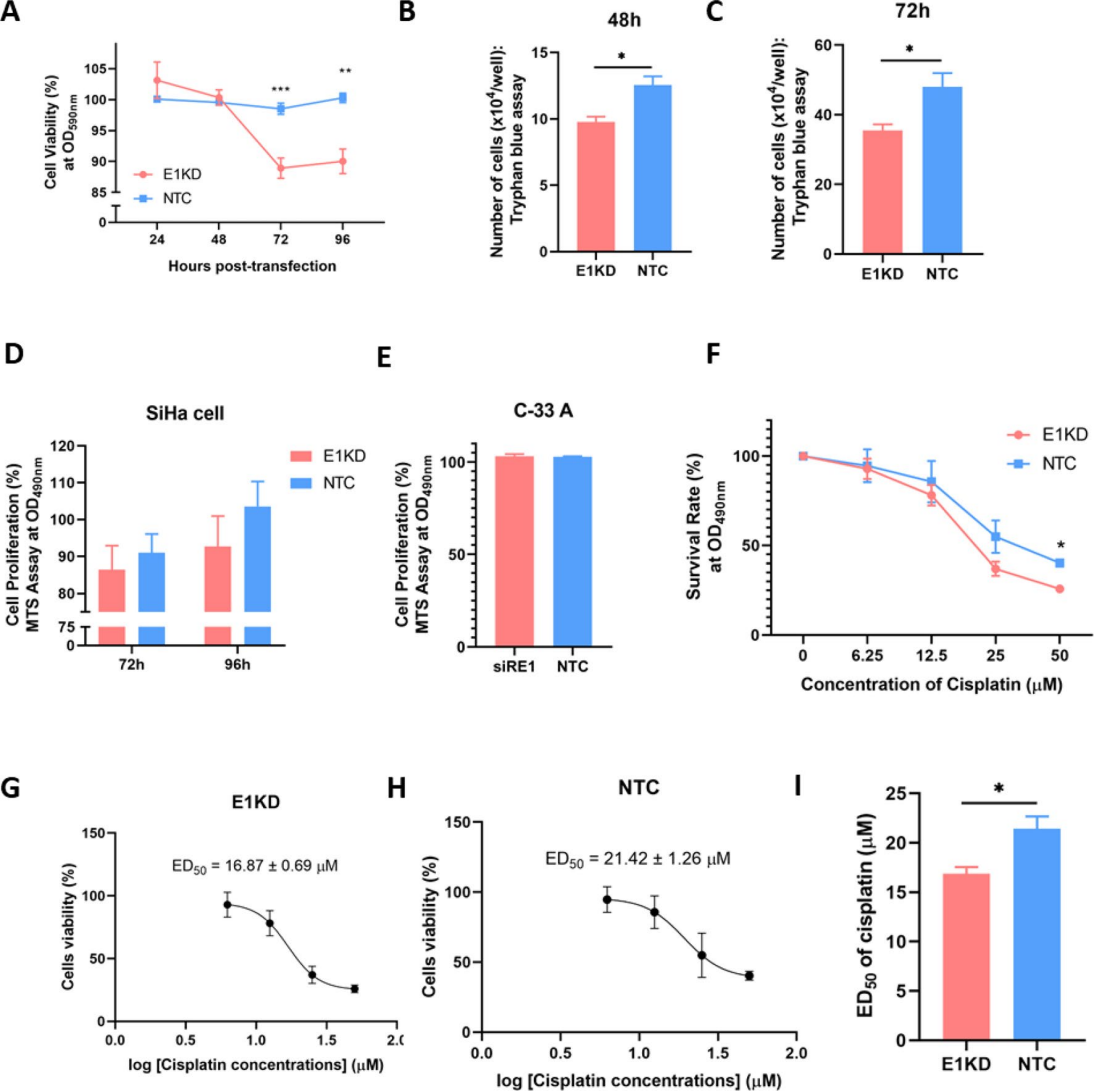
The cell growth properties of E1KD and NTC SiHa cell. (**A**–**D**) Cell growth properties were observed by different methods; (**A**) Crystal violet assay (**B**–**C**) Trypan blue cell count (**D**) MTS assay. (**E**) Percentage of cell proliferation in C-33 A cells transfected with siRE1.3–6 or NTC was accessed through the MTS assay. (**F**) Survival rate of E1KD SiHa cells after cisplatin treatments at various concentrations. (**G**–**I**) Median effective dose (ED50) of cisplation treatment in E1KD and NTC SiHa cells. Three independent experiments were conducted. Asterisks indicated a significant difference (*p < 0.05, **p < 0.01, ***p < 0.001*, unpaired t-test*). Error bars represent SEM.

P-FOXO3a localization plays a critical role in the control of cell growth^22^. Thus, the localization of P-FOXO3a was assessed by the immunofluorescence assay. E1KD and NTC SiHa cells were stained using rabbit polyclonal antibodies to FOXO3a (phospho S253), rhodamine phalloidin (actin staining), and DAPI (nuclear staining). In E1KD SiHa cells, the results revealed localization of P-FOXO3a (green) in both the nucleus (blue) and cytoplasm (red) (Fig. 2A). The percentage of nuclear P-FOXO3a in E1KD SiHa cells was higher than that in NTC SiHa cells (47.103 ± 5.827% vs. 6.983 ± 4.213%). Moreover, the correlation coefficient (R) showed a significant difference between E1KD and NTC SiHa cells (*p* = 0.0206). This indicated that E1KD SiHa cells frequently found P-FOXO3a nuclear localization.

### Downregulation of HPV16E1 altered cell viability and growth

E1KD and NTC SiHa cells at 24, 48, 72, and 96 h were determined for viability and proliferation. To evaluate cell viability and proliferation, Crystal Violet assay, Trypan Blue staining, and MTS assay were done. The results obtained from the crystal violet assay revealed that cell viability (%) between E1KD and NTC SiHa cells was the same at 24–48 h after transfection. At 72 and 96 h post-transfection, the cell viability of E1KD SiHa cells dropped, while NTC SiHa cells showed no change (Fig. 3A). Similar results were confirmed by standard trypan blue cell counts (Fig. 3B and C). The proliferation rate of E1KD SiHa cells obtained from the MTS assay was lower than that of NTC SiHa cells without a statistically significant difference (Fig. 3D). To confirm the effect of E1 on cell proliferation, C-33 A cells (without the HPV genome) were transfected with siRE1.3–6 and NTC for 72 h. The proliferation rate of those cells was the same (Fig. 3E). This finding suggests that siRE1.3–6 had no impact on cell proliferation and was specific to HPV16E1. Thus, the downregulation of HPV16E1 affects cell growth.

Due to cisplatin, a chemotherapy that is commonly used for cervical cancer treatment^23^, it is interesting to observe whether knocking down E1 affects the susceptibility of the cervical cancer cells. E1KD and NTC SiHa cells were exposed to various doses of cisplatin (0, 6.25, 12.5, 25, and 50 µM) for 48 h. The cell survival rate (%) was measured using the MTS assay. The result demonstrated that the sensitivity to cisplatin in both E1KD and NTC SiHa cells was dose-responsive. Interestingly, the survival rate of E1KD SiHa cells was significantly lower than that of NTC at 50 µM of cisplatin treatment (Fig. 3F). Additionally, it was discovered that the ED_50_ of cisplatin treatment between E1KD and NTC SiHa cells was significantly different (16.87 ± 0.69 vs. 21.42 ± 1.26 µM; *p* = 0.0391) (Fig. 3G–I).

### HPV16E1 downregulation had no impact on cell death properties

**Figure 4:**
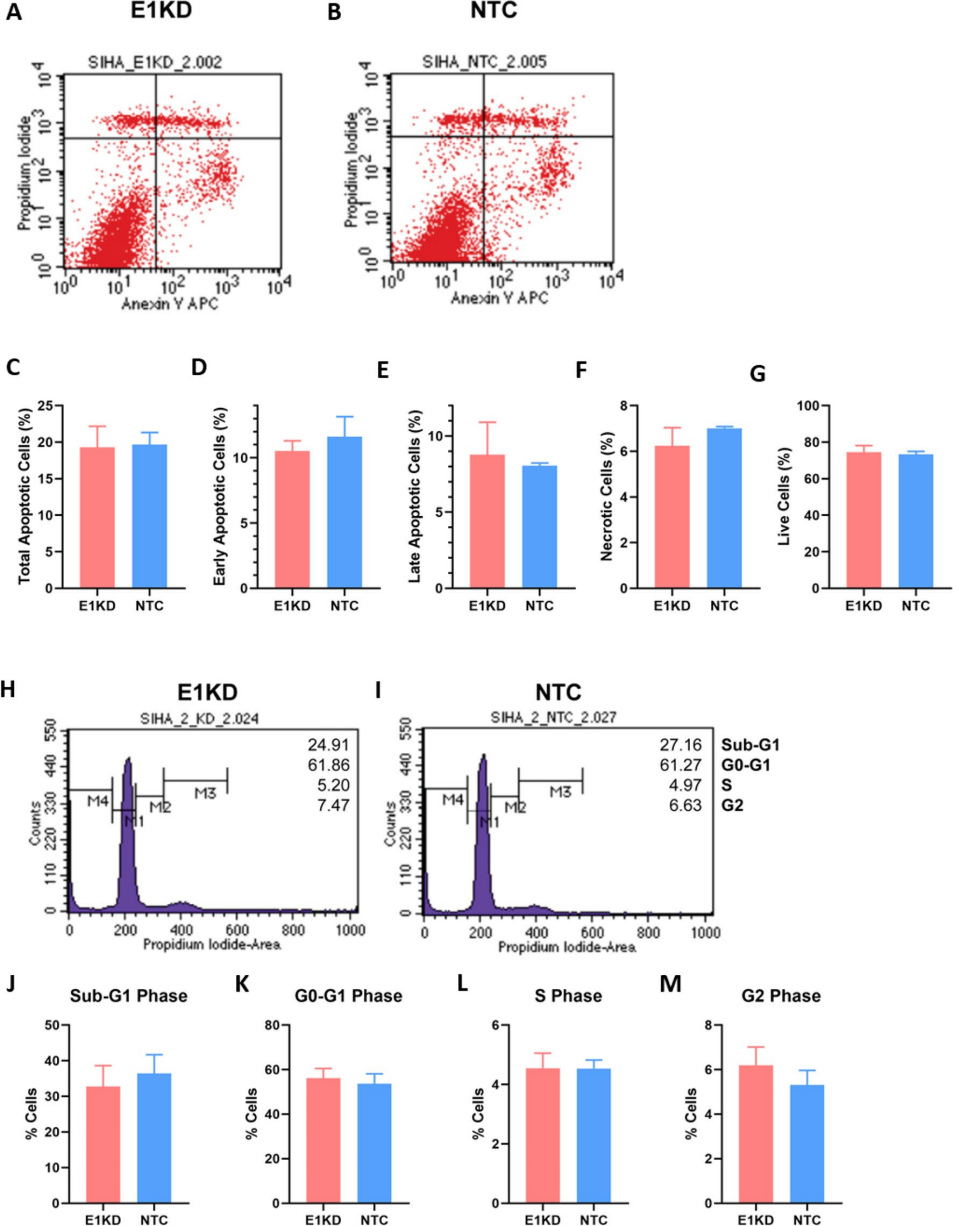
The population and cell cycle profiles of E1KD and NTC SiHa cell. (**A**,**B**) The scatter plot of apoptotic/necrotic populations in (**A**) E1KD and (**B**) NTC SiHa cell. (**C**–**G**) The percentage of (**C**) Total apoptotic (**D**) Early apoptotic (**E**) Late apoptotic (**F**) Necrotic (**G**) Live cells in each condition. (**H**,**I**) Flow cytometry analysis for cell cycle distribution of (**H**) E1KD and (**I**) NTC SiHa cells. (**J**–**M**) The percentage of the cells in each phase; (**J**) Sub-G1 (**K**) G0-G1 (**L**) S (**M**) G2 phase. Three independent experiments were conducted. Error bars represent SEM.

Since the viability of E1KD SiHa cells was lower than that of NTC SiHa cells (Fig. 3A–D), it is curious to know whether apoptosis or necrosis is involved in this phenomenon. Therefore, the E1KD and NTC SiHa cells were stained with annexin-V and PI and analyzed by flow cytometry. The percentages of each cell population (early-, late-, total apoptotic, and necrotic cells) in E1KD and NTC SiHa cells were the same (Fig. 4A–G). The mean fluorescence intensities (MFI) of annexin-V (apoptotic cells) and PI (necrotic and late apoptotic cells) were also the same under every circumstance (Supplemental Fig. 4). According to these results, the reduction of cell viability in E1KD SiHa cells (Fig. 3) might not be affected by apoptosis or necrosis activities. Then, the cell cycle profiles were analyzed. The proportions of E1KD SiHa cells and NTC SiHa cells in sub-G1, G0-G1, S phases, and G2 were comparable (Fig. 4H–M).

### Downregulation of HPV16E1 reduced metastatic property of cervical cancer cell

**Figure 5:**
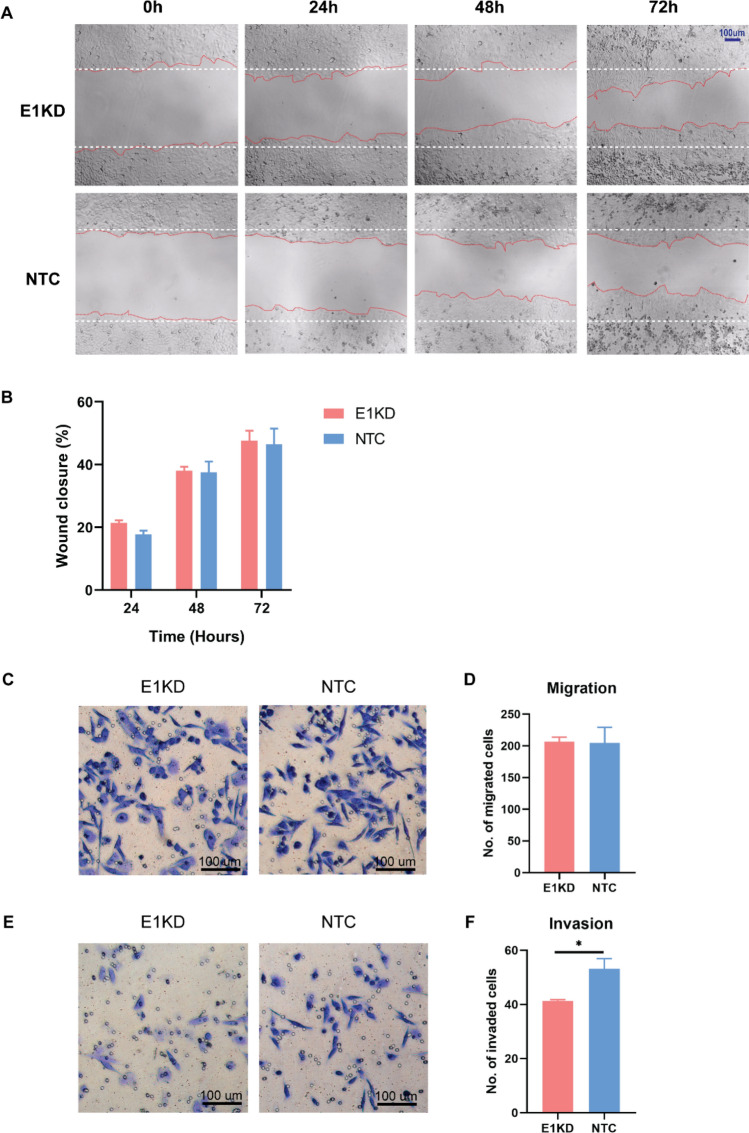
Cell migration and invasion properties in E1KD and NTC SiHa cells. (**A**) The wound healing assay. The cells were observed under an inverted microscope (40x). (**B**) Percentage of wound closure in each condition. (**C**) Transwell migration assay. (**D**) Number of migrated cells (**E**) Transwell invasion assay. (**F**) Number of invaded cells. The cells were counted under an inverted microscope (100x). Three independent experiments were conducted. Error bars represent SEM. Asterisks indicate a significant difference (*p < 0.05, *unpaired t-test*).

To explore whether HPV16E1 affects cancer cell properties, cell migration was determined by the wound healing assay. Following the scratching, the wound closure was checked every 24, 48, and 72 h. The results indicated that there was no significant difference between E1KD and NTC SiHa cells regarding the percentage of wound closure (Fig. 5A and B). To confirm these results, a transwell migration assay was performed. The findings revealed the same results (Fig. 5C and D). An attempt to investigate HPV16E1's function on cell invasion, a transwell invasion assay was conducted. When compared to NTC, E1KD SiHa cells had a significantly lower number of invaded cells (Fig. 5E and F). The expression of matrix metalloproteinases 9 (MMP9) and reversion-inducing cysteine-rich protein with kazal motifs (RECK) mRNA and protein were observed to confirm the impact of HPV16E1 on the association of cell invasion. However, there were no differences in the expression of MMP9 and RECK between E1KD and NTC SiHa cells (Supplemental Figs. 5, 6).

### HPV16E1 downregulation suppressed colony formation and anchorage-dependent cell growth

**Figure 6:**
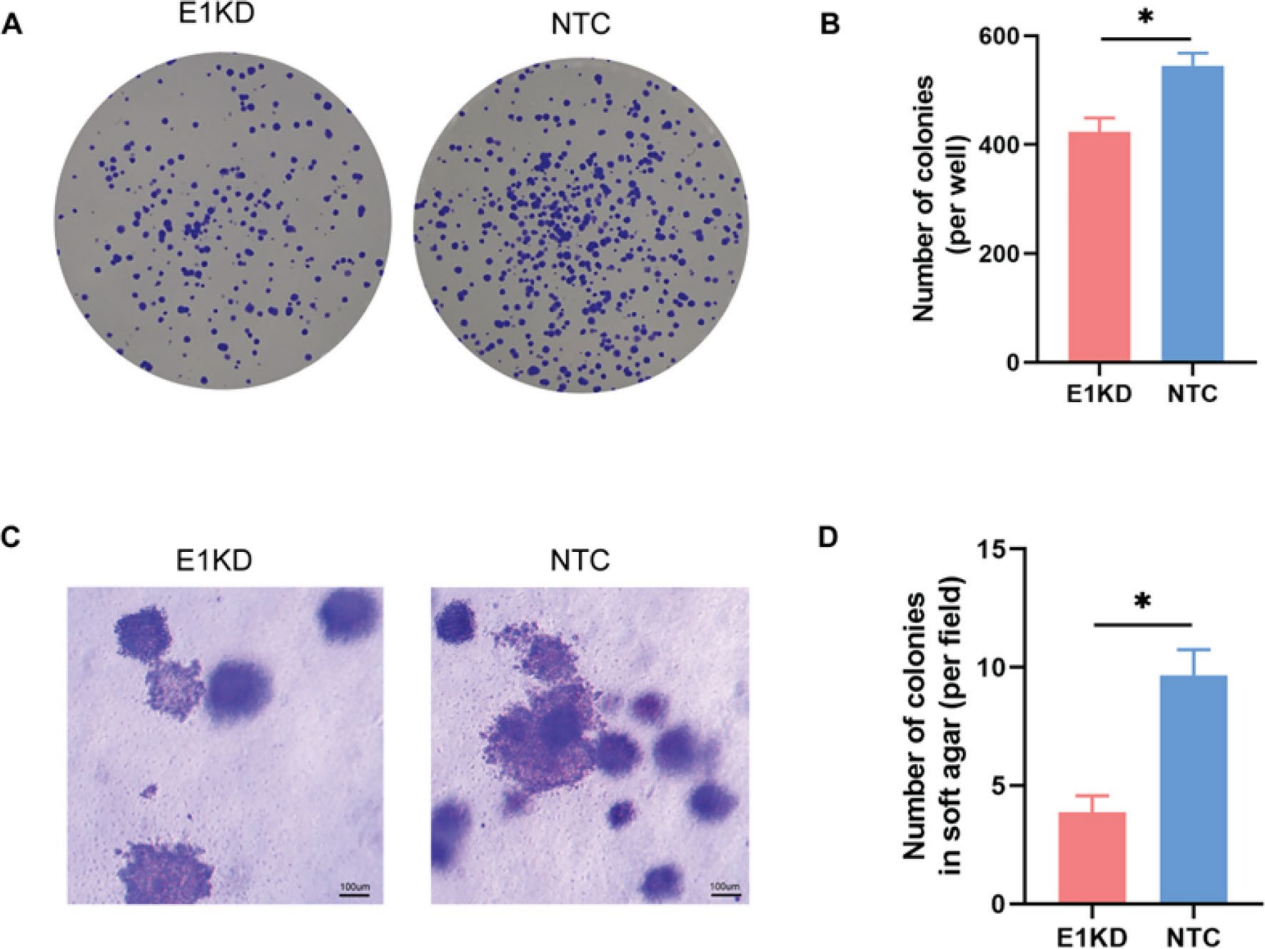
Colony formation in E1KD and NTC SiHa cells. (**A**) Clonogenic assay. The colonies were stained with 0.005% crystal violet and counted by image J software. (**B**) Number of colonies per well in the clonogenic assay. (**C**) Soft agar colony formation assay. The colonies were stained with 0.05% crystal violet and counted for five fields randomly. The cells were observed under an inverted microscope (40x). (**D**) Number of colony per field in soft agar. Three independent experiments were conducted. Asterisks indicate a significant difference (*p < 0.05, *unpaired t-test*). Error bars represent SEM.

The colony formation property of SiHa cells after HPV16 downregulation was observed by clonogenic assay. The number of colonies of E1KD SiHa cells was significantly lower than that of NTC SiHa cells (Fig. 6A and B). The ability of cancer-derived cells and transformed cells to survive and proliferate in the absence of anchorage to the extracellular matrix (ECM) and their surrounding cells is known as anchorage independence of growth, and it is highly correlated with tumorigenicity. Then, soft agar colony formation was performed. Interestingly, the number of E1KD SiHa cell colonies on the soft agar was significantly lower than that of NTC SiHa cells (Fig. 6C and D), corresponding to the clonogenic assay’s results.

## Discussion

HPV infection is the main risk factor for cervical carcinogenesis. Hr-HPV expresses E5, E6, and E7 that trigger the development of cervical cancer^24–26^. However, previous studies by our group demonstrated the possibility of HPV16E1 in cervical carcinogenesis^18,19,27^. In this study, E1 mRNA expression was knocked down in SiHa cells, an HPV16-positive cervical cancer cell, to determine the functions of HPV16E1 in cervical carcinogenesis.

Since HPV transcribes as polycistronic mRNA, knocking down only one gene may affect other HPV genes expression. Hence, the HPV16E6, E6*I, and E7 mRNA expressions were also analyzed in this study. Our findings demonstrated that E1 downregulation affected HPV16E6*I and E7 expression but not HPV16E6 (Fig. 1B–D). However, this phenomenon might not be affected by the activity of siRE1 since the sequence of siRE1.3–6 in this study was proven not to bind the major transcripts of HPV16E6*I and E7. In addition, the major transcript of HPV16E1 is monocistronic mRNA^12,13^. Thus, HPV16E7 expression should depend on the function of HPV16E1 protein but not HPV16E1 transcripts, which corresponds to the previous study indicating that HPV E1 protein can regulate the promoter that expresses the E6 and E7 mRNAs^21^. In contrast, E7KD SiHa cells did not affect E1 expression (Supplemental Fig. 1A). But when E7 mRNA expression was knocked down, E6 and E6*I expression were also downregulated (Supplemental Fig. 1B–D). Altogether, these results suggested that E1 expression is independent of both E6 and E7 expression, while E6*I and E7 expression may be influenced by E1.

HPV disrupted a number of host cellular pathways in order to trigger cervical carcinogenesis. The most prominent examples are the p53 and pRb degradation caused by Hr-HPV E6 and E7, respectively^24–26^. Moreover, in HPV16 E1 overexpressing human embryonic kidney (HEK) 293 T cells, FOXO3a was another host gene that was most downregulated^19^. This regulatory pathway is regulated by the PI3K/AKT pathway, which promotes cellular functions of cell growth and proliferation^28^. FOXO3a is inactivated by degradation through phosphorylation by P-AKT^29^, leading to an increase in cell survival signals^30^. In our study, only P-FOXO3a (S253) protein expression was altered when HPV16E1 was knocked down, whereas neither non-phosphorylated FOXO3a nor upstream signaling molecules (AKT and P-AKT) were changed (Fig. 1G). Moreover, it has been found that P-FOXO localization also plays a crucial role in the cellular mechanism. FOXOs are primarily found in the cytosol of numerous kinds of cancer as inactive phosphorylate forms^31,32^. On the other hand, upon localization of FOXO in the nucleus, it triggers the expression of genes related to cell cycle arrest and cell death^30^. In this study, FOXO3a localization was found to be regulated by HPV16E1 because FOXO3a was mostly localized in the nucleus of E1KD SiHa cells (Fig. 2). Additionally, FOXO1 localization in cervical cancer cells revealed a similar outcome. It was discovered that FOXO1 activation increased P-FOXO1 re-localization to the nucleus, which inhibited cell proliferation and induced apoptosis^32^. Our results suggested that in cervical cancer cells, HPV16E1 regulated the expression and localization of P-FOXO3a. The phosphorylation of FOXO3a has been reported to play a role in several cancers, such as breast, pancreatic, and kidney cancers^33–36^, which may also contribute to cervical cancer development. The presence of nuclear P-FOXO3a (S253) suggested that cell characteristics, particularly cell growth, would be altered. According to this hypothesis, the effect of P-FOXO3a regulated by HPV16E1 on cell growth, apoptosis, and other properties, e.g., metastasis and anchorage-independent properties, were further investigated.

In this study, we demonstrated that HPV16E1 affected cell viability. A significant reduction in cell viability was observed in E1KD SiHa cells (Fig. 3A–C), which corresponded to the declining proliferation rate’s result (Fig. 3D). These findings suggested that HPV16E1 may contribute in some way to the viability or proliferation of cancer cells. It is possible that E1 directly regulates the FOXO signaling pathway. The regulation of FOXO on cell growth was mentioned in some studies. Nephroblastoma cell growth was inhibited by the overexpression of FOXO3a^37^. FOXO1 reactivation reduced the proliferation rate of SiHa and HeLa cells^32^. In addition, there was a possibility that the alteration of cell growth in HPV16E1KD SiHa cells might be an indirect effect of HPV16E7 downregulation since E7 was also downregulated in E1KD SiHa cells (Fig. 1D and G). It is well known that HPVE7 promotes cell proliferation through pRb inhibition and degradation^38^.

It is interesting to see whether the susceptibility of E1KD cells changes or not after treatment with chemotherapy medicine. One of the most effective chemotherapy drugs for treating cervical cancer is cisplatin^39^. In this study, HPV16E1 downregulated cervical cancer cells increased cisplatin susceptibility (Fig. 3F). It is noted that cisplatin interferes with DNA replication mechanisms in order to induce cytotoxic effects^39^. When compared to the primary function of HPVE1, which promotes viral DNA replication^40^, this function is the opposite. Therefore, cisplatin may interfere with the function of E1. The rate of DNA replication in E1KD SiHa cells treated with cisplatin may be extremely low, resulting in a decrease in the cell survival rate of E1KD SiHa cells. In addition, it has been found that enhancing nuclear FOXO1 improved the efficacy of cisplatin in reducing tumor volume in an ovarian cancer xenograft model^41^. Moreover, overexpression of FOXO3a enhanced the apoptotic rate in cisplatin-resistant ovarian cancer cells^42^. Hence, it is possible that the improvement of cisplatin susceptibility in E1KD SiHa cells (Fig. 3F) was also involved in the FOXO3a signaling pathway, including expression (Fig. 1F) and localization (Fig. 2).

Cell death (apoptosis and necrosis) and the alteration of cell cycle profiles are essential mechanisms that control the growth of cells. Apoptosis and necrosis are the two main subtypes of programmed cell death. While apoptotic cells quietly die without releasing their contents into the environment, necrotic cells release their contents into the environment, which triggers inflammatory reactions^43^. In this study, when HPV16E1 was downregulated, neither the apoptotic nor necrotic cell populations were altered (Fig. 4A–G). In contrast, our previous research showed that overexpressing HPV16E1 in HEK-293 T caused an increase in cell apoptosis^19^. It is possible that the excess of E1 in HPV16E1-overexpressing HEK-293 T cells caused DNA damage, leading to an increase in apoptosis. Many previous studies have shown that E7 has an impact on cell apoptosis. The HPV-16E7 protein increased the expression of the inhibitor of apoptosis protein (IAP), which prevented TNF-mediated apoptosis in healthy human fibroblasts^44,45^. On the other hand, E7 may cause cell apoptosis in primary human keratinocytes, according to some evidence^46^. Even though E7 was reduced in the E1KD SiHa cells (Fig. 1D and G), the cell death property might not have been affected. Other HPV oncogenes, particularly HPV16E6, which was previously known as the anti-apoptotic protein that causes p53 degradation, are present in SiHa cells^47^. The possibility of HPVE6 functions on E1KD SiHa cells could not be ruled out. According to the findings, it appeared that neither necrotic nor apoptotic pathways were involved in the decrease in cell viability and proliferation in E1KD cells.

The cell cycle decision is one of the most crucial processes in cell growth. A cell spends the majority of its time among the interphase (G1, S, and G2 phases). The cell exits interphase, enters mitosis, and begins to divide when the growth signal is activated. On the other hand, when a dysregulation is detected, such as when DNA damage occurs, the cell cycle is stopped in order to aid in the repair process. There was no difference in the percentage of E1KD SiHa cells in the sub-G1, G0-G1, S, and G2 phases (Fig. 4H–M). These results suggested that HPV16E1 might not be involved in the control of the cell cycle.

The term "metastatic cancer" describes cancer that has spread to areas of the body other than the primary tumor site. Previous research showed that the WNT/-catenin signaling pathway promoted cell migration by downregulating FOXO3a in the cervical cancer cells, HeLa and Ca Ski^48^. In addition to cervical cancer, nephroblastoma cells (a rare kidney cancer that primarily affects children; Wilms' tumor) were found to exhibit reduced cell migration due to overexpression of FOXO3a^37^. Additionally, there was a significant correlation between FOXO3 and the metastasis of a number of cancers, including breast, pancreatic, and kidney cancers^33–36^. Unexpectedly, our findings did not detect any effects of HPV16E1 on cell migration properties (Fig. 5A–D). Therefore, the E1 function may not be involved in the cell migration property. In the case of HPV infection, both in vitro and in vivo studies have shown that HPV16E6 encourages cervical cancer cell migration^49^. In this study, the expression of the HPV16E6 oncogene was not downregulated by E1KD (Fig. 1B). As a result, E1KD cells should have the same migration characteristics as NTC cells. Cell invasion properties in E1KD cells were further evaluated. The results revealed that the invaded properties of the cells were altered when HPV16E1 was downregulated (Fig. 5E and F), suggesting that HPV16E1 function is related to cell invasion. Numerous studies revealed that either FOXO3a or HPV16E7 contributed to cell invasion^48,49^. In HeLa and MDA-MB-435 cells (a human breast cancer cell line), FOXO3a encourages cell invasion through the induction of matrix metalloproteinases (MMPs)^50^. It has been shown that overexpressing FOXO3a encourages cell invasion in some cancers, such as glioblastoma and gastric cancer^51,52^. Taken together, although it has no effect on cell migration, HPV16 E1 may contribute to the invasion of cervical cancer cells. The possibility that HPV16E1 might control key enzymes like MMPs, which are crucial for breaking down the ECM. To confirm this hypothesis, the expression of MMP9, one of the key types of MMPs usually involved in ECM digestion, was observed. Unexpectedly, there was no relationship between MMP9 expression and HPV16E1 downregulation (Supplemental Fig. 5A and C). In addition, the expression of Reversion-inducing cysteine-rich protein with kazal motifs (RECK), a natural inhibitor of MMP9, was also not changed after HPV16E1 downregulation (Supplemental Fig. 5B and D). These results indicated that MMP9 might not be involved in this phenomenon. However, there are other MMPs that are responsible for ECM digestion and cell invasion. There is a need for further studies. Moreover, it has been shown that Suprabasin (SBSN), a novel oncogene encoded by humans^53^, was upregulated in an HPV16E1 overexpressing cell^19^. This protein has been identified as an oncoprotein in esophageal cancer and highly invasive glioblastoma^54^. It is possible that HPV16E1 might associate with this protein to promote cell invasion. Future studies need to be conducted to confirm this association.

One characteristic of cancer cells is their capacity to multiply into colonies from single cells. Interestingly, when HPV16E1 was downregulated, the number of colonies significantly and dramatically decreased (Fig. 6A and B). The reduction in the number of colonies of SiHa and CaSki cells was demonstrated in nuclear receptor related-1 protein (Nurr1), a transcriptional factor that is regarded as an early response gene and is activated by a variety of signals, such as stress and growth factors^55^, downregulation condition after γ-irradiation exposure. It is interesting to conduct a future study to observe the relationship between HPV16E1 and Nurr1 in the clonogenic property of cervical cancer cells.

Anchorage-independent growth is the ability of cancer-derived cells and transformed cells to persist and multiply in the absence of anchorage to the ECM and their surrounding cells. This characteristic exhibits a strong correlation with tumorigenicity^56^. The anchorage-independent growth of SiHa cells was affected by the E1KD condition (Fig. 6C and D). E1KD SiHa cells showed less colony formation than control cells. This suggested that anchorage-independent cell growth was suppressed by downregulating HPV16E1. The role of FOXO in anchorage-independent cell growth has been reported. Upregulation of FOXO3a inhibited anchorage-independent cell growth in MCF7 breast cancer cells^57^. While FOXO1 was downregulated in U87 human glioblastoma cells, which led to an increase in the number of colonies on soft agar. Additionally, one of the most highly expressed genes in the HPV16E1-overexpressing HEK 293 T cells was glucocorticoid kinase-1 (SGK1)^19^. It's interesting to note that activation of SGK1 has been shown to be sufficient to promote anchorage-independent growth in soft agar. As a result, the loss of the anchorage-independent growth property in E1KD SiHa cells may be related to SGK1 expression; however, no research on SGK1 expression in E1KD SiHa cells has been demonstrated. Future research ought to be done.

**Figure 7:**
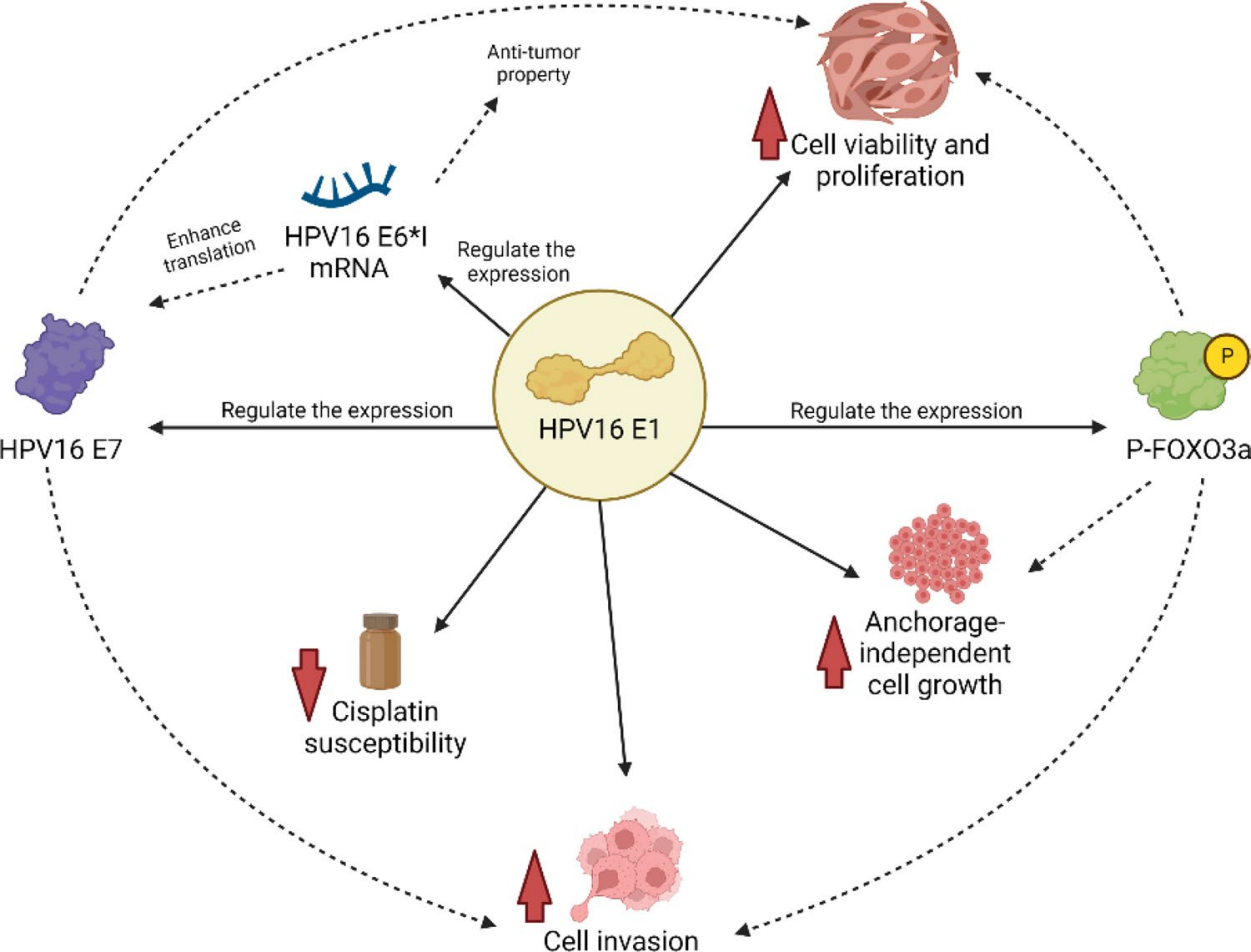
The possible role of HPV16E1 in the regulation of cancer cell properties*.* The expression of P-FOXO3a, E7, and E6*I was regulated by HPV16E1, resulting in the regulation of cell viability/proliferation, invasion, and anchorage-independent cell growth properties. In addition, E1 influenced the cisplatin susceptibility of cervical cancer cells (solid line). The possible mechanisms (dotted line) involved in controlling the cancer cell properties were the functions of HPVE7, E6*I, or human FOXO3a.

In conclusion, this study showed that HPV16E1 downregulation impacts P-FOXO3a and HPV16E7 expression. E1 downregulation also affected some essential properties of cancer cells, including cell viability, colony formation, cisplatin susceptibility, invasion, and anchorage-independent cell growth. Nevertheless, the expression of an essential oncogene, HPV16E7, was also downregulated in E1KD SiHa cells. Because of the synergistic effect of E7 downregulation along with E1 downregulation, all of the changed properties in E1KD SiHa cells might be affected either indirectly by E7 together with E1 or directly by E1. The suggested role of HPV16E1 in cervical carcinogenesis according to this study is summarized in Fig. 7. Further study should be conducted to find more evidence about the role of HPV16E1 in cervical carcinogenesis. The protein target of HPV16E1 in the regulation of the FOXO pathway is under investigation; this might be an interaction between E1 and P-FOXO3a directly or an alternative pathway such as the interaction with serum and glucocorticoid-regulated kinase (SGK)^30^. Therefore, the role of HPV16E1 in the FOXO signaling pathway should be explored in future studies. However, this study strongly demonstrated that HPV16E1 affects the properties of cancer cells, whether through direct or indirect effects. Hence, these findings provided a new potential role for HPV16E1 in cervical carcinogenesis.

## Methods

### Cell lines

SiHa cells (EP-CL-0210), an HPV16-positive (1–2 copies per cell) cervical cancer cell line, were obtained from Elabscience Biotechnology (USA). These cells were cultured in Modified Eagle Medium (MEM; Gibco, USA) supplemented with 10% fetal bovine serum (FBS) (Gibco, USA), 0.01 M HEPES, 0.2% NaHCO_3_, 100 units penicillin, 100 µg streptomycin, and grown under 5% CO_2_ at 37 °C.

C-33 A cells, cervical cancer cells without HPV infection, were cultured in Dulbecco's Modified Eagle Medium (DMEM; Cat no. SH30022.02, Hyclone, USA) supplementary with 10% FBS (Gibco, USA), 100 units penicillin, 100 µg streptomycin, and grown under 5% CO_2_ at 37 °C.

### siRNAs transfection

The siRNA targeting HPV16E1 mRNAs was designed using the web-based online siDESIGN center (Horizon, UK) (https://horizondiscovery.com/en/ordering-and-calculation-tools/sidesign-center), and synthesized by Dharmacon (UK) with ONTARGETplus modification. The siRNA sequences were shown in Supplemental Table 1. For transfection, 1 × 10^5^ SiHa cells/well (in a 24-well plate) or 7 × 10^3^ SiHa cells/well (in a 96-well plate) were grown overnight at 37 °C in 5% CO_2_ to reach 60–80% confluency. Prior to transfection, the cells were washed with 1X PBS, and the 10% MEM growth medium (500 µL/well for a 24-well plate or 100 µL/well for 96-well plate) was added. Then, the siRNA mixture was transfected into the cells by Lipofectamine RNAiMAX Reagent (Invitrogen, USA), using a standard protocol provided by the manufacturer (final concentration = 50 µL for a 24-well plate; 10 µL for a 96-well plate). E1 knockdown (E1KD) cells were incubated for the specified number of days in each experiment (1–4 days) at 37 °C with 5% CO_2_. Cell scrapers were used for the RT-qPCR and western blot experiments, while cell trypsinization was used for the rest to harvest the cells for subsequent experiments. ON-TARGETplus Non-targeting Control#1 siRNAs (Dharmacon, UK) were used as a negative control (NTC).

To silence the expression of HPV16E6 and E7, SiHa cells were transfected with either HPV16E6 siRNA (sc-156008; Santa Cruz Biotechnology, USA), HPV16E7 siRNA (sc-270423; Santa Cruz Biotechnology, USA), HPV16E6 + E7 siRNA, or NTC to a final concentration of 5 pmol/well by using a similar protocol as described above.

### Real-time PCR

RNA from each condition was extracted through the RNeasy mini kit (Qiagen, Germany). The purity and yield of the extracted RNA were accessed by the NanoDrop (Eppendrof, USA). After that, first-stranded cDNA was synthesized by SuperScriptTM III Reverse Transcriptase (Invitrogen, USA) using 200 ng of RNA input. The cDNA construction reaction was performed by following a standard protocol provided by the manufacturer. Then, 2 µL cDNA samples were amplified by 2X SsoAdvanced Univer-sal SYBR Green Supermix (Bio-Rad, USA), and specific primers (0.25 nM/reaction) (Supplemental Table 2), using QuantStudio™ 5 Real-Time PCR System (Applied Biosystems, USA). The real-time PCR conditions were 95 °C for 30 s, followed by 40 cycles of 95 °C for 15 s and 60 °C for 60 s. The expression of each mRNA was calculated by double delta Ct analysis using GAPDH as an internal control.

### SDS-PAGE and western blot

The E1 knockdown (KD) SiHa cells (72 h of 50 nM siRE1.3–6 transfected cells) were harvested using Cell Scrapers and then collected by centrifugation at 300 × *g* for 5 min. The supernatant was discarded. The sample was mixed with 80 µL of Lysis M (Roche, Germany) by vortexing for 10 s. The cells were then incubated for another five min at room temperature. After that, the protein sample (30 µg/well for FOXO3a, P-FOXO3a, HPV16E7, and GAPDH; 50 µg/well for AKT and P-AKT; 200 µg/well for MMP-9 and RECK) from whole cell lysate was 1:1 mixed with SDS loading buffer (5.3% of 2 mercaptoethanol in 2X Laemmli sample buffer; BIO-RAD, USA), and heated at 95 ºC for 5 min using ProFlex™ Thermal Cycler (Applied biosystems, USA). Then, the protein was separated by SDS-PAGE and western blot using the protocol previously described^58^. The blots were cut prior to hybridization with antibodies. 1:1000 primary antibody, Phospho-Akt (Ser473) (587F11) Mouse monoclonal antibody (mAb) #4051 (Cell Signaling Technology, USA), recombinant rabbit mAb anti-FOXO3a antibody ab109629 (Abcam, USA), mouse anti-MMP9 antibody (E-11) sc-393859 (Santa cruz biotechnology, USA), or mouse anti-GAPDH antibody sc-47724 (Santa cruz biotechnology, USA), diluted in 1% BSA in TTBS were used as primary antibody by incubating at 4 ºC overnight, whereas 1:15,000 secondary antibody, goat polyclonal antibody to mouse IgG conjugated with horseradish peroxidase (HRP) or Goat polyclonal Antibody to rabbit IgG conjugated with horseradish peroxidase (HRP) (Abcam, USA) diluted in 1% BSA in TTBS were used as secondary antibody by incubating for one hour at room temperature. The target band was developed by SuperSignal^®^ West Femto Maximum Sensitivity Substrate (Thermo Scientific, USA) and observed by Chemidoc XRS + (BIO-RAD, USA). The band intensity was measured using ImageJ software version 1.53t. Three independent experiments were conducted. For the re-probing protocol, the membrane was washed with StripPRO 1 Min Stripping Buffer (Visual protein, Taiwan) by using the standard protocol from the manufacturer. Then, the membranes were probed with 1:1000 of primary antibody (either rabbit anti-FOXO3a (phospho S253) antibody ab47285 (Abcam, USA), Akt Rabbit mAb #4685 (Cell Signaling Technology, USA), or mouse anti-RECK antibody sc-373929 (Santa cruz biotechnology, USA),). The subsequent procedure was carried out in the same manner.

### Immunofluorescence assay

SiHa cells (7 × 10^3^ cells) were grown overnight at 37 °C with 5% CO_2_ in the 8-well slide chamber (Lab-Tek, USA) and transfected with 50 nM siRE1.3–6 using Lipofectamine RNAiMax reagent. After 72 h of transfection, the cells were washed and fixed in cold acetone, and then blocked for an hour with 1% BSA in 1X PBS at room temperature. The air-dry slide was then stained with 1:100 rabbit anti-FOXO3a (phospho S253) antibody and 1:20 rhodamine phalloidin (Invitrogen, USA) mixture, at 4 °C overnight in a moisture chamber. After that, the slide was incubated with a 1:1000 goat polyclonal antibody to rabbit IgG conjugated with FITC (#ab6717; Abcam, USA) for another hour at 37 °C. Then, the slide was stained with 300 nM DAPI solution (#422801; Biolegend, USA) for 5 min at room temperature. Then, the slide was treated with anti-fade fluorescence mounting medium before being observed under the 63 × magnification of the LSM800 airy scan confocal microscope (ZEISS, Germany). The FOXO-positive cells were counted for five fields in each condition to observe the localization. Prism8.0.1 software was used to calculate the Pearson correlation coefficient (r) from the total number of cells and the cell containing nuclear FOXO3a. Three independent experiments were conducted.

### Crystal violet assay

siRE1.3–6 transfected SiHa cells in a 96-well plate at 24, 48, 72, and 96 h were stained with 100 µL 0.1% crystal violet solution for 30 min. Then, the plate was washed with distilled water (DW) and air-dried at room temperature. To elute pre-stained crystal violet, 100 µL 10% acetic acid (Merck, Germany) in DW was added to each well. The absorbance was measured at 590 nm using a VICTORTM X3 microplate reader (Perkin Eimer, USA). The number of live cells was determined by using the viable cell standard curve, which has been constructed from various concentrations of SiHa cells (1 × 10^4^ to 7 × 10^4^ cells/well). Three independent experiments were done.

### Trypan blue assay

Trypsinization was used to harvest the 48- and 72-h E1KD and NTC SiHa cells from the 24-well plate. Trypan blue (Invitrogen, USA) and cell suspension were combined in an equal volume (1:1) before being loaded onto a hemocytometer (Spencer, USA). The cells were then examined using a microscope in four areas (16 squares = 1 area).

### Cell proliferation assay (MTS)

The proliferation rate of siRE1.3–6 and NTC transfected SiHa and C-33A cells was observed at 72-, and/or 96-h post-transfection by mixing with 10 µL CellTiter 96^®^ Aqueous One Solution Cell Proliferation Assay (Promega, USA), incubating at 37 ºC for one hour, and measuring at an absorbance of 490 nm using a microplate reader. Three independent experiments were conducted.

### Cisplatin treatment

At 48 h post-transfection, the E1KD and NTC SiHa cells were incubated for an additional 48 h with 100 µL of cisplatin (#P4394, Sigma-aldrich, USA) diluted in 10% MEM growth medium at various concentrations (50, 25, 12.5, 6.25, and 0 µM). The MTS assay was applied to measure the survival rate of the cells following cisplatin treatment. ED50 was calculated by Prism8.0.1 software. Three independent experiments were done.

### Flow cytometry

To observe the population of apoptotic cells, 1 × 10^6^ 72-h E1KD and NTC SiHa cells were stained by APC Annexin V and propidium iodide (PI) (Biolegend, USA). The procedures were performed according to the manufacturer’s instructions. For the cell cycle stage, the 72-h E1KD and NTC SiHa cells were treated with 100 µg/mL of RNase A, DNase, and protenase-free (Thermo Fisher Scientific, USA) before staining with propidium iodide (PI). The method was followed according to the manufacturer’s instructions. The stained cells were analyzed by BD FACSCalibur flow cytometry (BD Biosciences, USA). Three independent experiments were done.

### Wound healing assay

1 × 10^5^ cells/well of 48-h E1KD and NTC SiHa cells were seeded onto the 24-well plate and cultured for 24 h. A sterile 200 µL pipette tip was used to make the wound on the cell monolayer. The cells were then continued in culture with a new medium at 37 °C and 5% CO_2_. The wound width was measured at 0, 24, 48, and 72 h under 4 × magnification of the inverted microscope ECLIPSS TS100 (Nikon, Japan) 6 fields/well using PixitPro A4 software (LANOPTIK Technologies, China). The percentage of wound closure was calculated. Three independent experiments were conducted.

### Cell migration and invasion assay

The protocol was adapted from Justus C.R. et al. 2014^59^. Briefly, the 48-h E1KD SiHa cells were harvested from the 24-well plate by trypsinization. Then, E1KD SiHa cells (1 × 10^5^ cells/well) in serum-free MEM were seeded onto each cell insert, a transparent PET membrane 24-well with a 8.0 µm pore size (Falcon, USA). Next, the cells were incubated at 37 °C for 10 min, and complete 10% MEM (growth medium) was added to the lower well. After 24 h of incubation at 37 °C, cell insert was removed, and non-migrated cells were removed by the cotton-tipped applicator. After being fixed with 70% ethanol, the migrated cells were then stained with a 0.2% crystal violet solution and washed by dipping in water. Under an inverted microscope, 5 fields/well of migrated cells were counted. Three separate tests were conducted.

The cell invasion assay was performed by adding extracellular matrix (ECM) from Engelbreth-Holm-Swarm murine sarcoma (#E6909; Sigma-aldrich, USA) to each insert at a final concentration of 250 µg/mL. Then, the plate was incubated at 37 °C for 2 h to allow the gel to solidify. Next, the cells were added to the insert in the same manner of the transwell migration assay.

### Colony formation (clonogenic) assay

500 cells/3 mL of 48-h E1KD and NTC SiHa cells were seeded onto a 6-well plate, which was then cultured at 37 °C with 5% CO_2_ in a moisture chamber. The colonies were stained after incubating for 11 days with 0.005% crystal violet. ImageJ software quantified the colony counts. Three independent experiments were conducted.

### Soft agar colony formation assay

The protocol was adapted from Borowicz S. et al. 2014^60^. In brief, 0.5% agar–agar (Millipore, USA) in 10% MEM was added to the 6-well plate (1.5 mL/well) to create the bottom layer of agar. Then, the agar was allowed to solidify for 30 min at room temperature. To prepare the upper layer of agar-containing cells, 1.5 mL of a mixture of 1 × 10^4^ cell/well of E1KD/NTC SiHa cells and 0.3% agar–agar in 10% MEM was added. The agar was allowed to solidify at room temperature for another 30 min. The plate was then incubated for 21 days in a moisture chamber at 37 °C, 5% CO_2_ with continuous adding 10% MEM twice a week. The colonies were stained with 0.05% crystal violet solution. Five fields of colonies per well were counted using an inverted microscope. Three independent experiments were done.

### Statistics

Data were shown as the mean ± standard error of mean (SEM) and analyzed by *unpaired t-test* using GraphPad Prism version 7. *p* < 0.05 was considered as statistically significance.

### Ethical statement

This study was approved by the Institutional Review Board of the faculty of Medicine, Chulalongkorn University, a WHO-certified ethics committee (COA No.364/2020, IRB No. 042/60, 27 January 2020 and COA No.0051/2023, IRB No. 0035/66, 18 January 2023). The protocol of study was also approved by the Institutional Biosafety Committee (IBC) of the faculty of Medicine, Chulalongkorn University (MDCU-IBC018/2019 and MDCU-IBC006/2023).

### Supplementary Information

Supplementary Information.

## Data Availability

The data presented in this study are available upon reasonable request from the corresponding author.
