# Supplementary material for: HPV16E1 downregulation altered the cell characteristics involved in cervical cancer development
Source: Sci Rep. 2023 Oct 25;13:18217. doi: 10.1038/s41598-023-45339-1 (PMC10600143; doi:10.1038/s41598-023-45339-1)
Supplement: Supplementary file 1 — Supplementary Information. [file 41598_2023_45339_MOESM1_ESM.pdf]

HPV16E1 downregulation altered the cell characteristics involved in cervical cancer development

Thanayod Sasivimolrattana [1, 2], Arkom Chaiwongkot [2,3], and Parvapan Bhattarakosol [2,3,*]

[1] Medical Microbiology Interdisciplinary Program, Graduate School, Chulalongkorn University, Bangkok 10330, Thailand
[2] Center of Excellence in Applied Medical Virology, Department of Microbiology, Faculty of Medicine, Chulalongkorn University, Bangkok 10330, Thailand
[3] Division of Virology, Department of Microbiology, Faculty of Medicine, Chulalongkorn University, Bangkok 10330, Thailand
[*] Correspondence: parvapan.b@chula.ac.th or bhparvapan@gmail.com

Supplementary Figures and Tables

Supplemental Table 1. The siRNA targeting HPV16 E1 mRNAs was designed using the web-based online siDESIGN center (Horizon, UK), and synthesized by Dharmacon (UK) with ONTARGETplus modification.

Name	siRNAs sequence (21 nt; 5' to 3')	Start position
siRE1.3	Sense: 5' GUGGAUGUGUAGACAAUAAUU 3'<br>Antisense: 5' PUUAUUGUCUACACAUCCACUU 3'	296
siRE1.4	Sense: 5' GGAGGUGAUUGGAAGCAAAUU 3'<br>Antisense: 5' PUUUGCUUCCAAUCACCUCCUU 3'	1306
siRE1.5	Sense: 5' GGGAUGUAAUGGAUGGUUUUU 3'<br>Antisense: 5' PAAACCAUCCAUUACAUCCCUU 3'	39
siRE1.6	Sense: 5' AGGACGAGGACAAGGAAAAUU 3'<br>Antisense: 5' PUUUUCCUUGUCCUCGUCCUUU 3'	1871

Supplemental Table 2. The specific primers for RT-qPCR

Target	Sequences (5' to 3')	References
HPV16E1	F: GCGGGTATGGCAATACTGAA<br>R: TAACACCCTCTCCCCCACTT	(Bogovac et al., 2011)
HPV16E6	F: CGACGTGAGGTATATGACTTTGC<br>R: AGGACACAGGACACAGTGGCTTTTGACA	(Baedyananda et al., 2017)
HPV16E6*I	F: ACTGCGACGTGAGGTGTATTAAC<br>R: TGGAATCTTTGCTTTTTGTCC	(Chaiwongkot et al., 2020)
HPV16E7 FL	F: CAGCTCAGAGGAGGAGGATG<br>R: GCCCATTAACAGGTCTTCCA	(Chaiwongkot et al., 2020)
FOXO3a mRNA	F: TTCAAGGATAAGGGCGACAGCAAC<br>R: TGCCAGGCCACTTGGAGAG	(Kannike et al., 2014)
MMP9 mRNA	F: CCTGCCAGTTTCCATTCATC<br>R: GCCATTCACGTCGTCCTTAT	(Schröpfer et al., 2010)
RECK mRNA	F: TGCAAGCAGGCATCTTCAAA<br>R: ACCGAGCCCATTTCATTTCTG	(Zhou et al., 2015)
GAPDH mRNA	F: GAGTCAACGGATTTGGTCGT<br>R: TTGATTTTGGAGGGATCTCG	(Joseph et al., 2012)

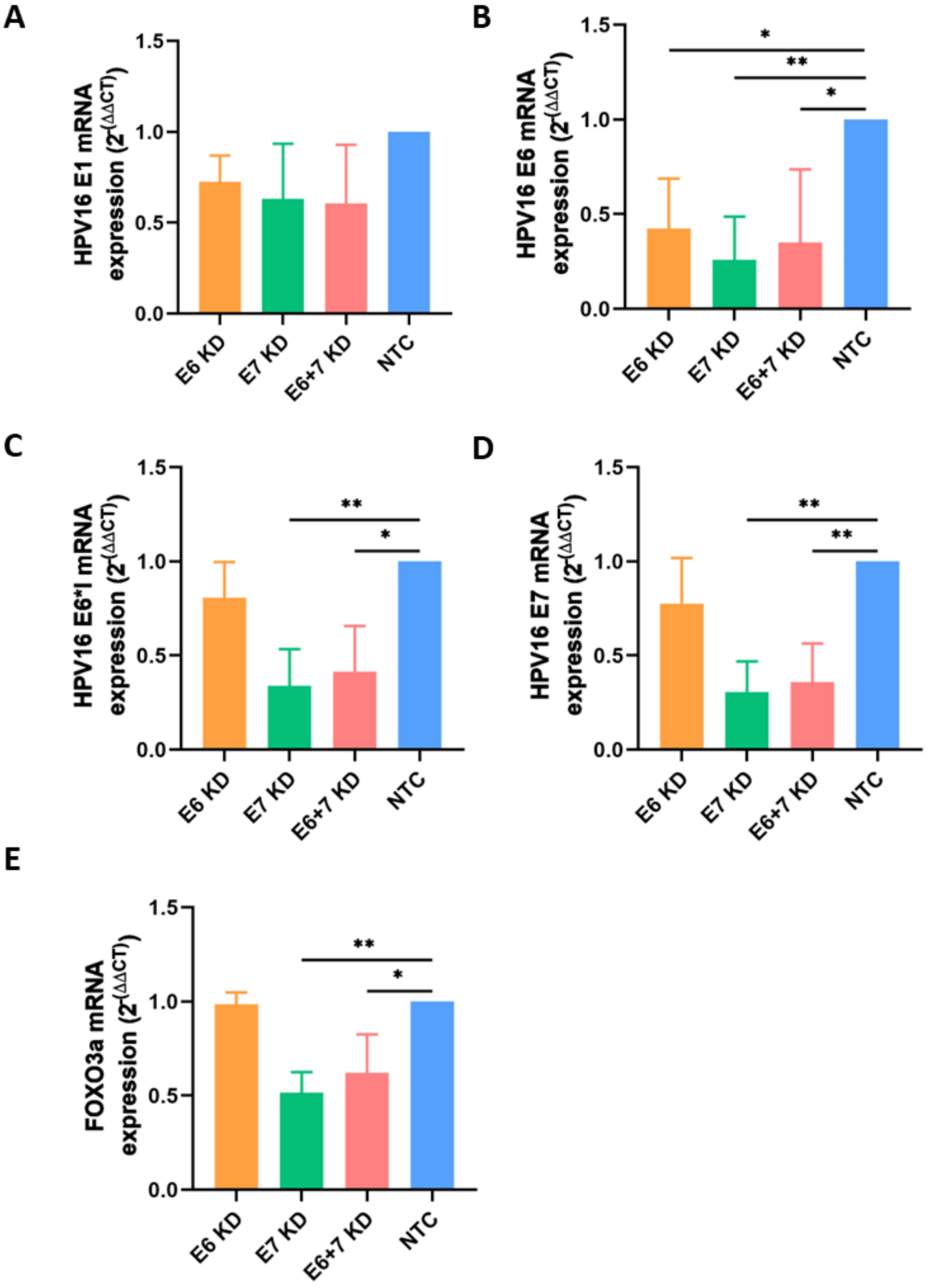

Supplemental Figure 1. HPV16 E1, E6, E6*I, E7, and FOXO3a mRNA expression in E6KD, E7KD, and E6+E7KD cells. Three independent experiments were conducted. Asterisks indicated a significant difference (*$p < 0.05$, **$p < 0.01$, *unpaired t-test*). Error bars represent SEM. GAPDH was used as an internal control.

(A)

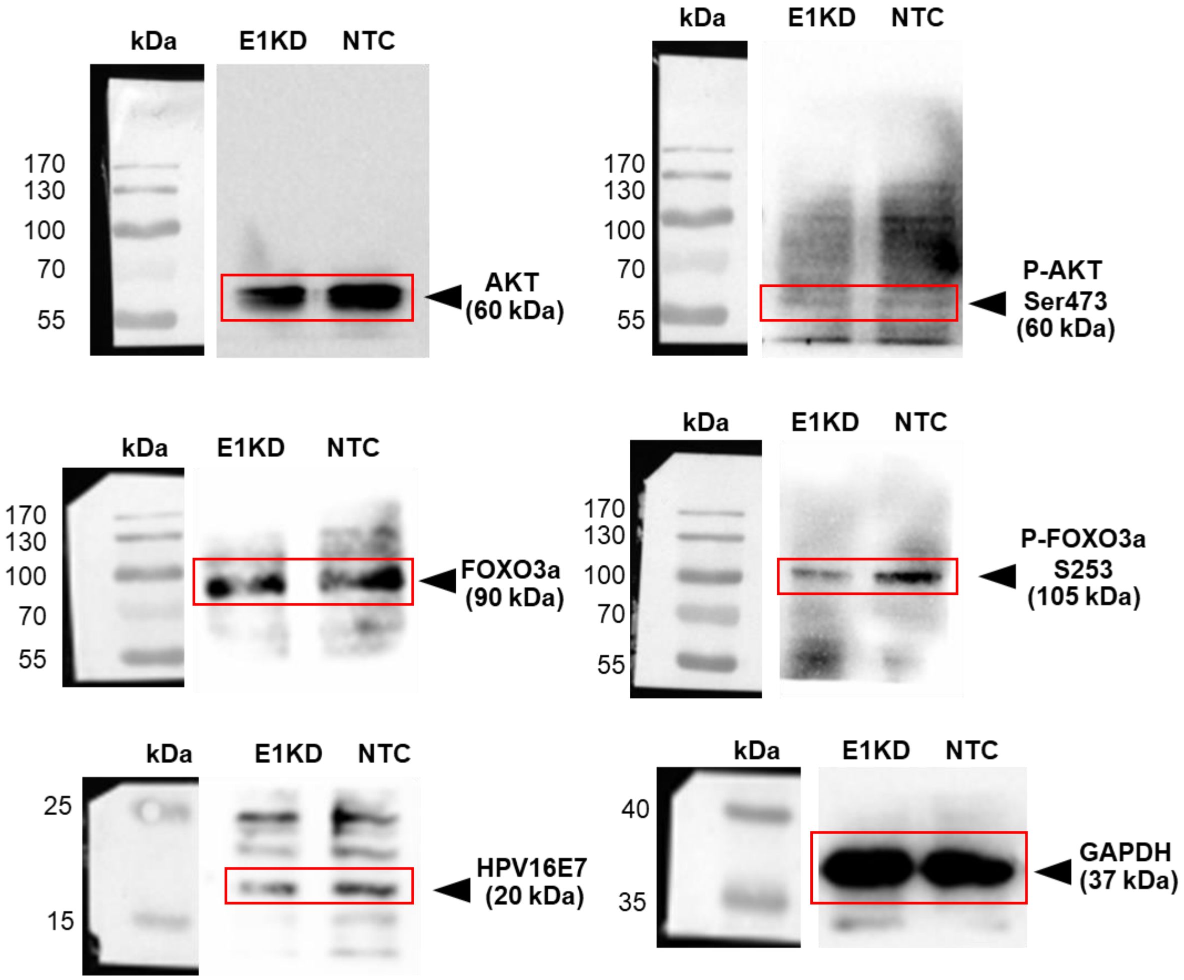

Supplemental Figure 2. Whole blots from SDS-PAGE and Western blot analysis of AKT, P-AKT, FOXO3a, P-FOXO3a, HPV16E7, and GAPDH proteins are shown in Figure 1F. Red boxes indicate the cropped portion of each immunoblot presented in Figure 1F. The blots were cut prior to hybridization with antibodies. The ladder and protein band were observed by Chemidoc XRS+ (BIO-RAD, USA); the protein ladder was captured by colorimetric method, whereas the protein band was captured by chemiluminescence method. Three independent experiments were performed as shown in A-C.

(B)

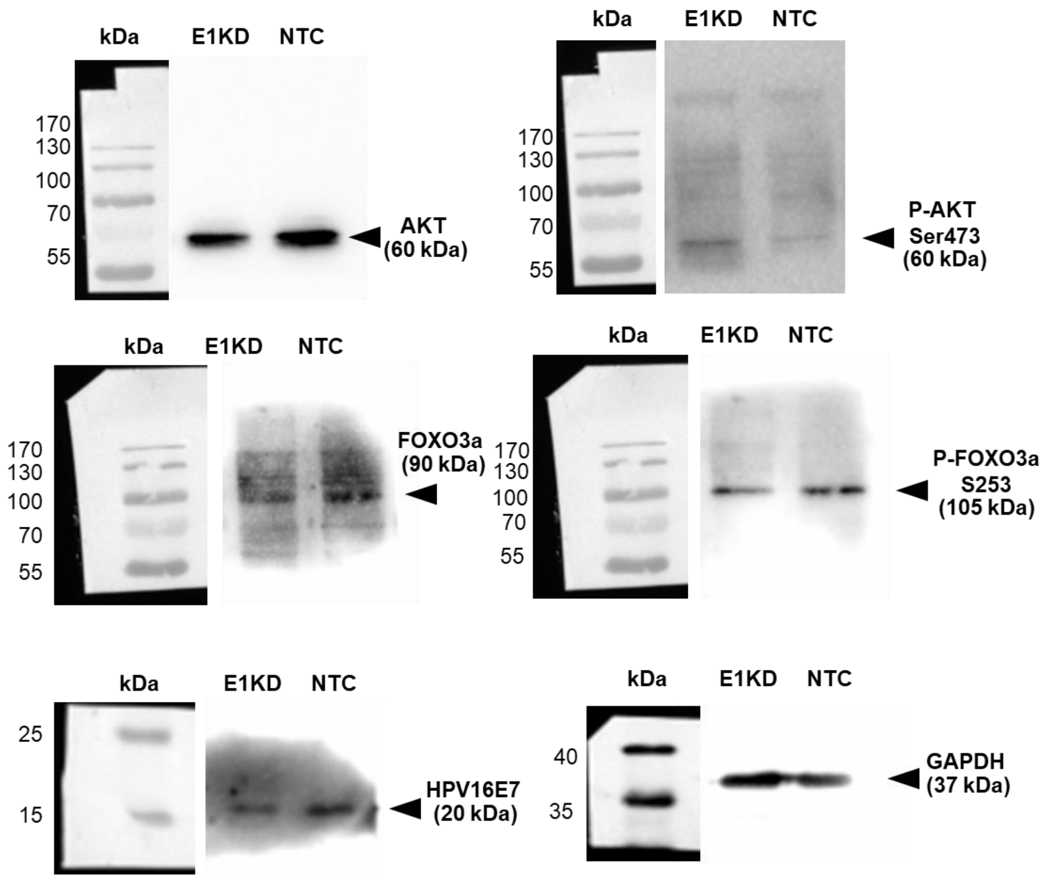

Supplemental Figure 2 (continued). Whole blots from SDS-PAGE and Western blot analysis of AKT, P-AKT, FOXO3a, P-FOXO3a, HPV16E7, and GAPDH proteins are shown in Figure 1F. Red boxes indicate the cropped portion of each immunoblot presented in Figure 1F. The blots were cut prior to hybridization with antibodies. The ladder and protein band were observed by Chemidoc XRS+ (BIO-RAD, USA); the protein ladder was captured by colorimetric method, whereas the protein band was captured by chemiluminescence method. Three independent experiments were performed as shown in A-C.

(C)

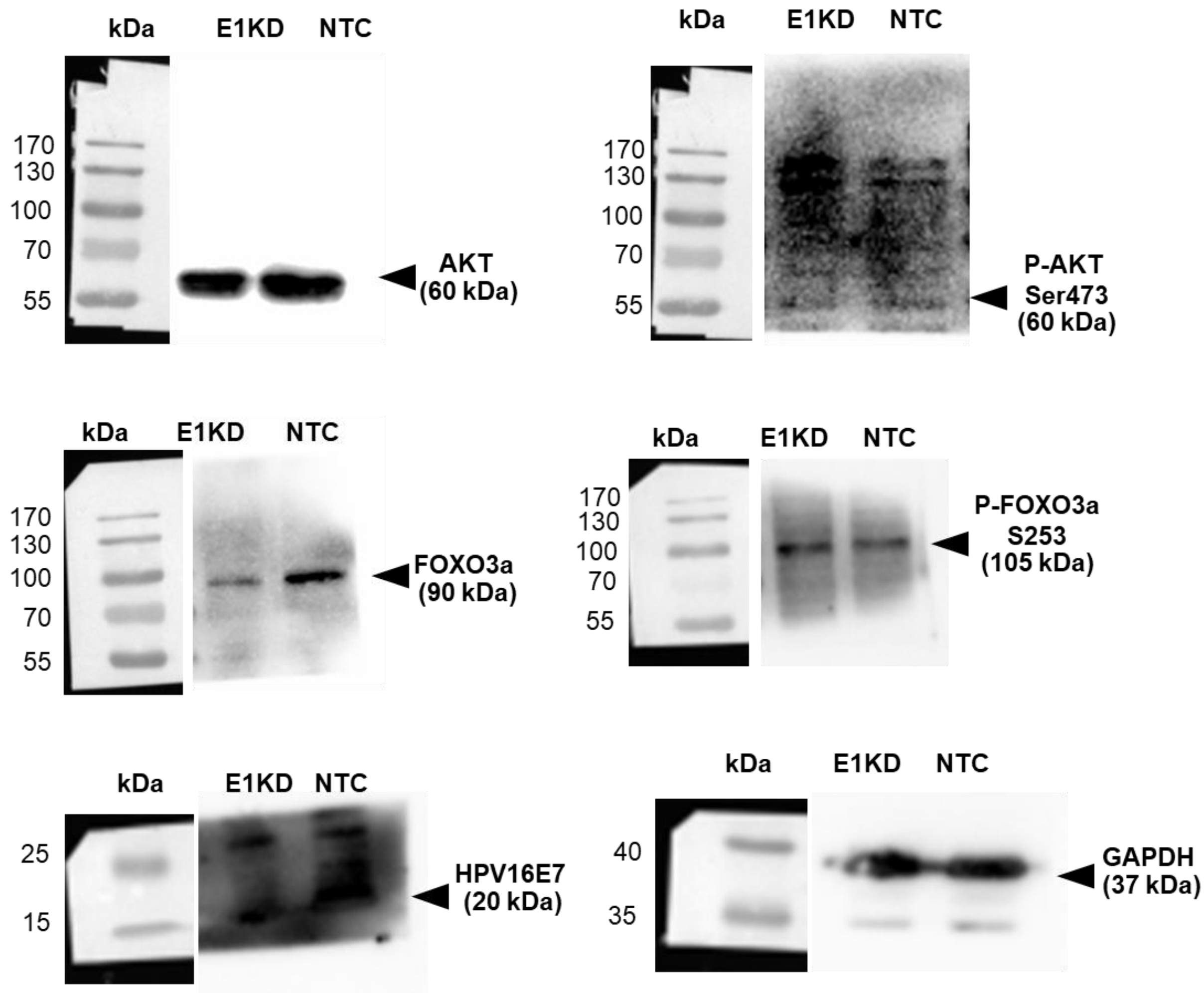

Supplemental Figure 2 (continued). Whole blots from SDS-PAGE and Western blot analysis of AKT, P-AKT, FOXO3a, P-FOXO3a, HPV16E7, and GAPDH proteins are shown in Figure 1F. Red boxes indicate the cropped portion of each immunoblot presented in Figure 1F. The blots were cut prior to hybridization with antibodies. The ladder and protein band were observed by Chemidoc XRS+ (BIO-RAD, USA); the protein ladder was captured by colorimetric method, whereas the protein band was captured by chemiluminescence method. Three independent experiments were performed as shown in A-C.

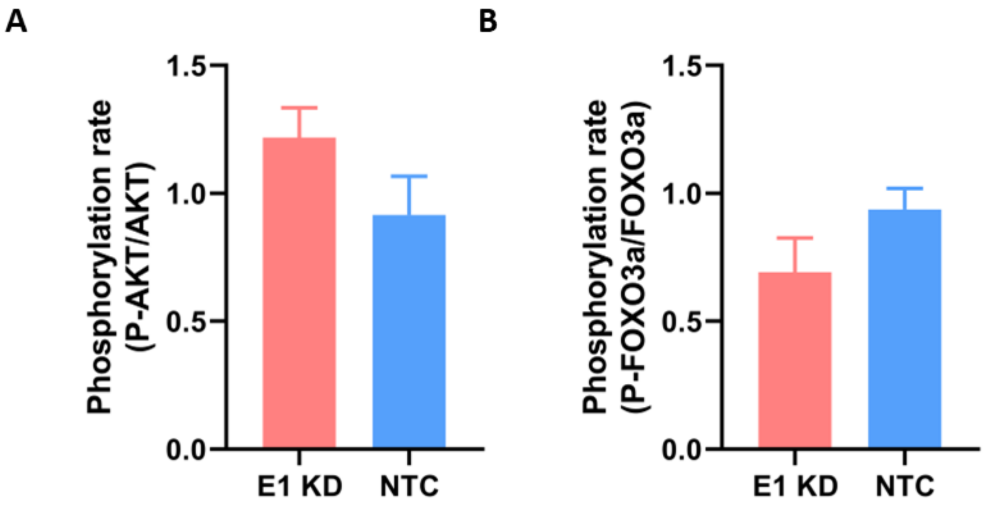

Supplemental Figure 3. The phosphorylation rate of AKT and FOXO3a in E1KD SiHa cells **(A)** P-AKT **(B)** P-FOXO3a. Three independent experiments were conducted. An *unpaired t-test* was used to calculate the significant difference. Error bars represent SEM.

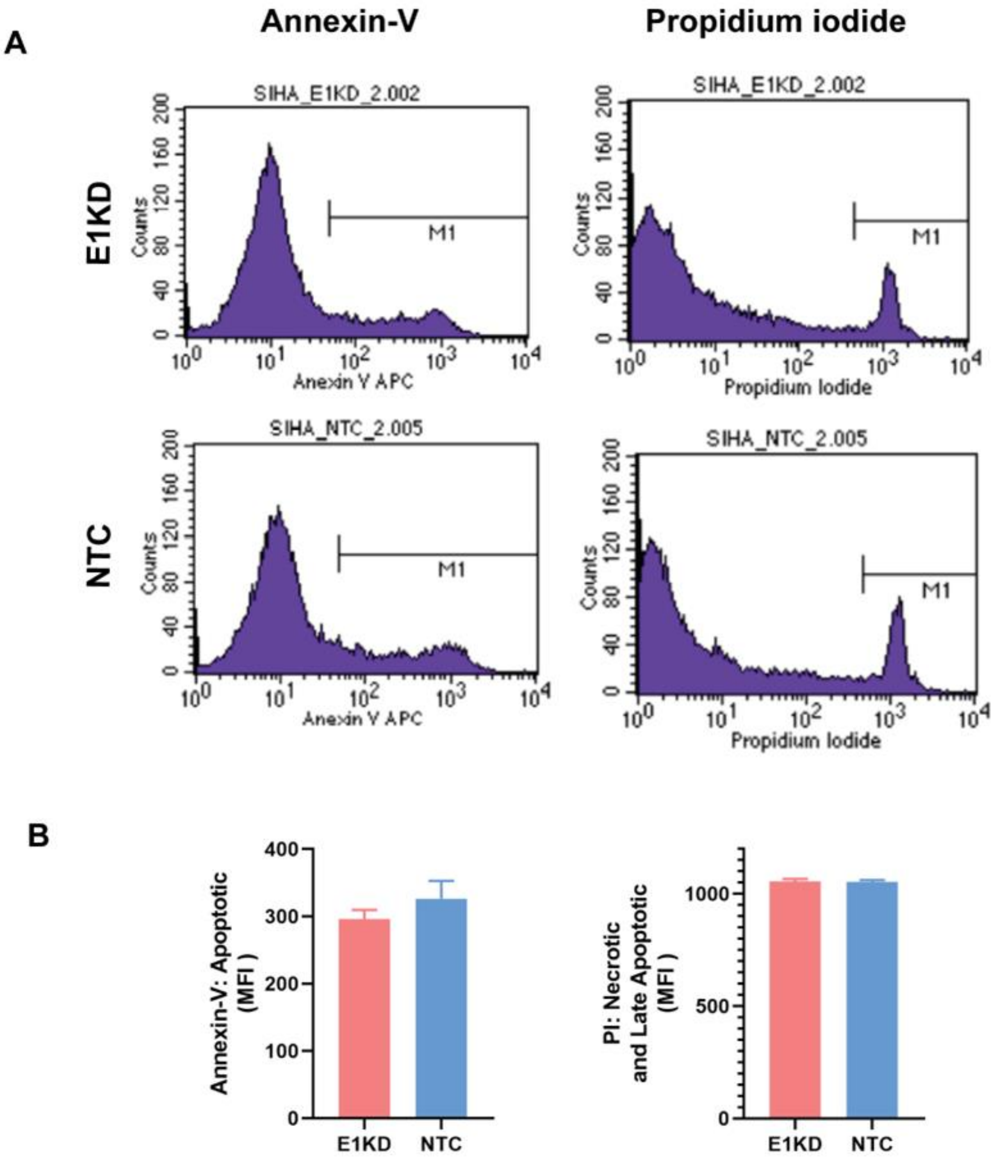

Supplemental Figure 4. The mean fluorescence intensity (MFI) analysis by flow cytometry. The E1KD and NTC cells were stained with Annexin-V and Propidium iodide. **(A)** Histogram represented the MFI of PI and annexin V. **(B)** The MFI of annexin-V (apoptosis) and PI (necrosis and late apoptosis). Three independent experiments were conducted. An *unpaired t-test* was used to calculate the significant difference. Error bars represent SEM.

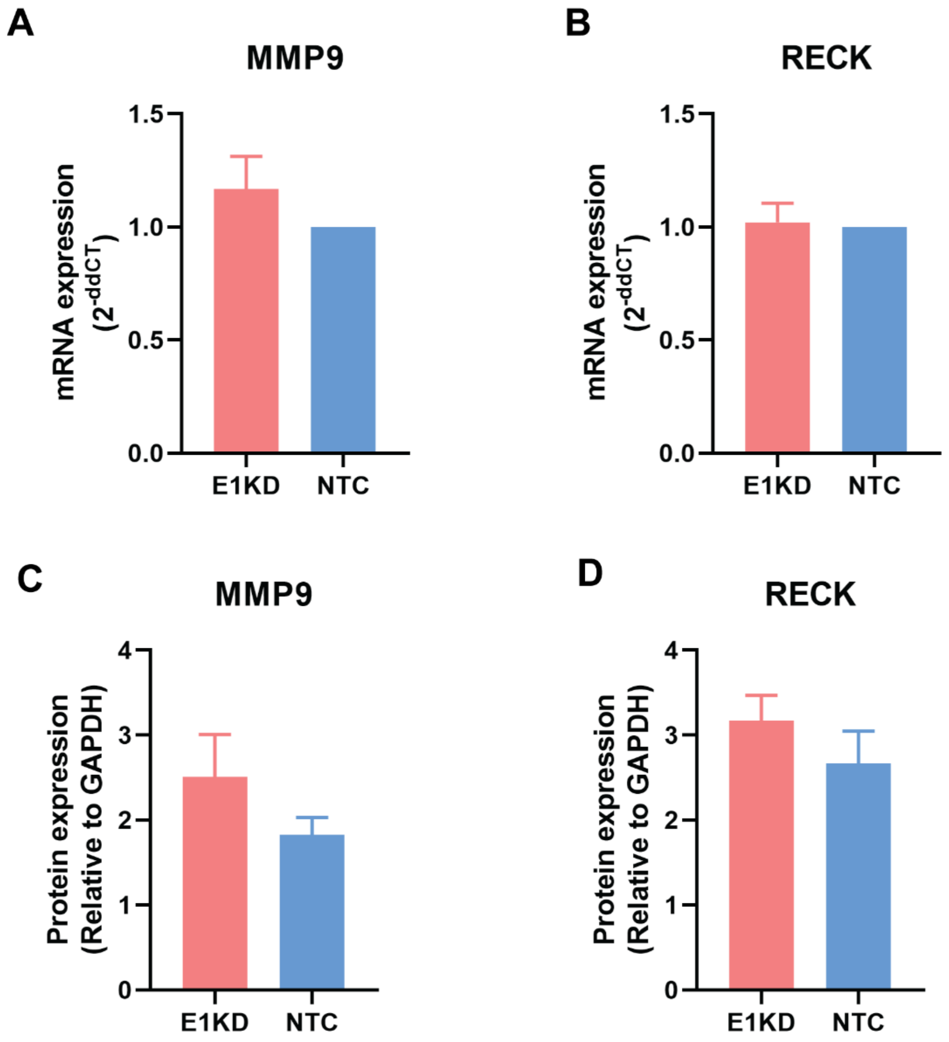

Supplemental Figure 5. The expression of MMP9 and RECK. (A-B) mRNA expression was measured by RT-qPCR; (A) MMP9 (B) RECK. (C-D) Protein expression were observed by western blot; (C) Relative expression of MMP9 (D) Relative expression of RECK. An unpaired t-test was used to calculate the significant difference. Error bars represent SEM.

(A)

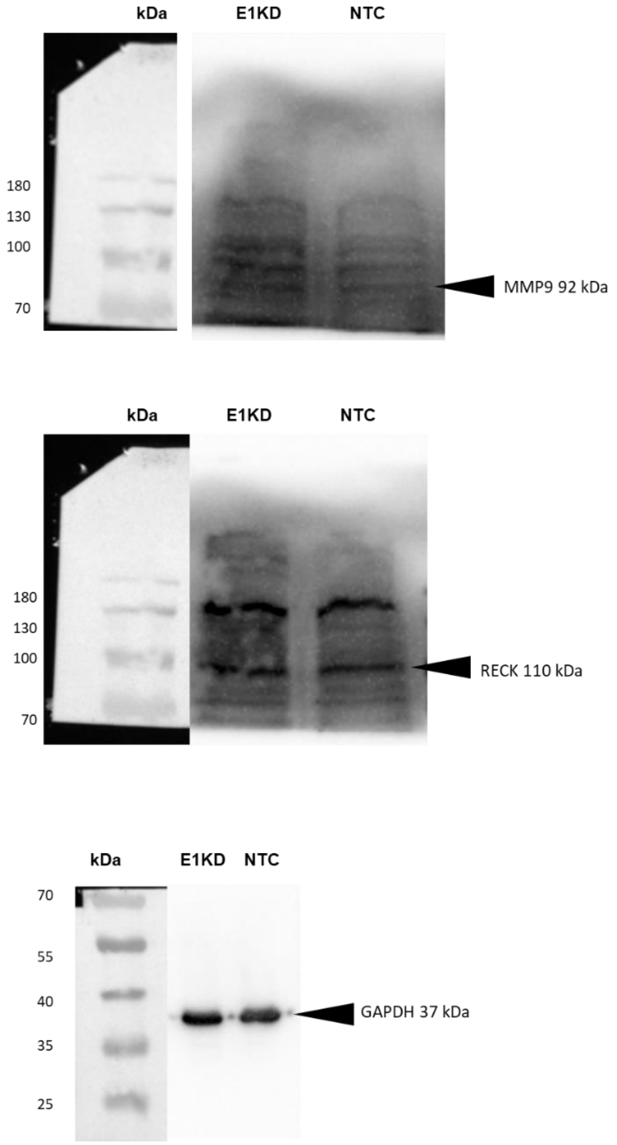

Supplemental Figure 6. Whole blots from SDS-PAGE and Western blot analysis of MMP9, RECK, and GAPDH. The blots were cut prior to hybridization with antibodies. The ladder and protein band were observed by Chemidoc XRS+ (BIO-RAD, USA); the protein ladder was captured by colorimetric method, whereas the protein band was captured by chemiluminescence method. Three independent experiments were performed as shown in A-C.

(B)

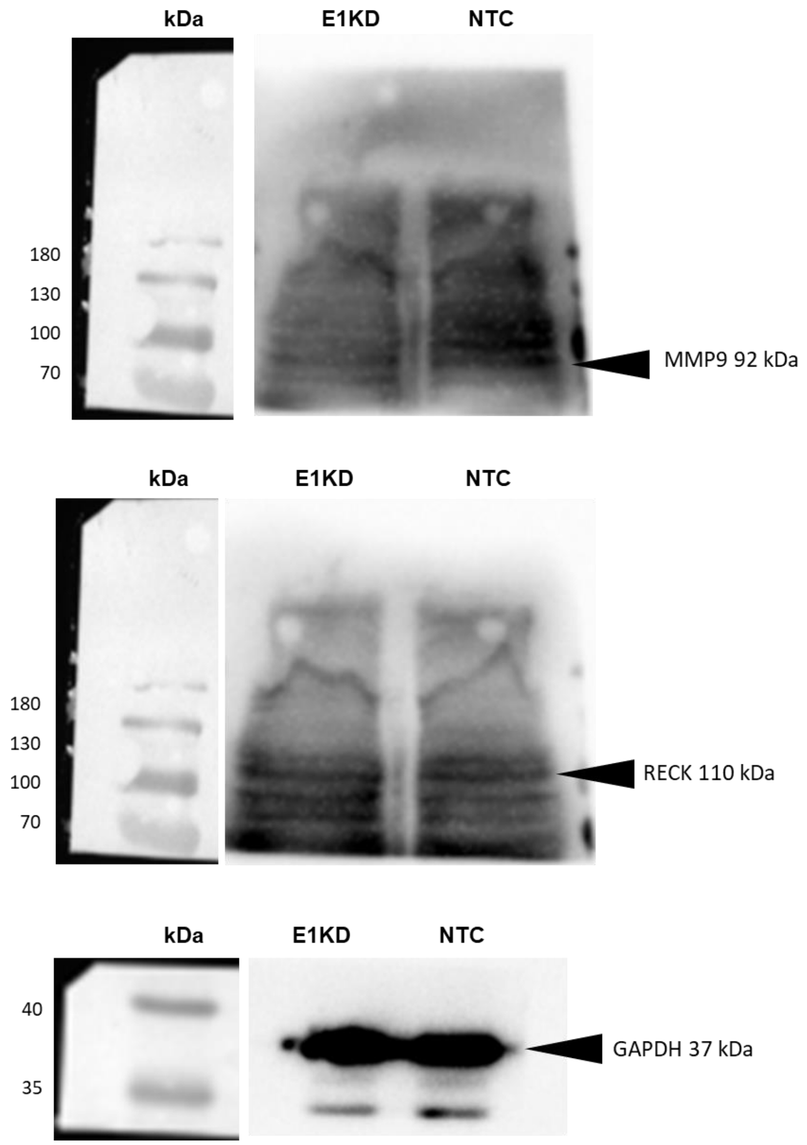

Supplemental Figure 6 (continued). Whole blots from SDS-PAGE and Western blot analysis of MMP9, RECK, and GAPDH. The blots were cut prior to hybridization with antibodies. The ladder and protein band were observed by Chemidoc XRS+ (BIO-RAD, USA); the protein ladder was captured by colorimetric method, whereas the protein band was captured by chemiluminescence method. Three independent experiments were performed as shown in A-C.

(C)

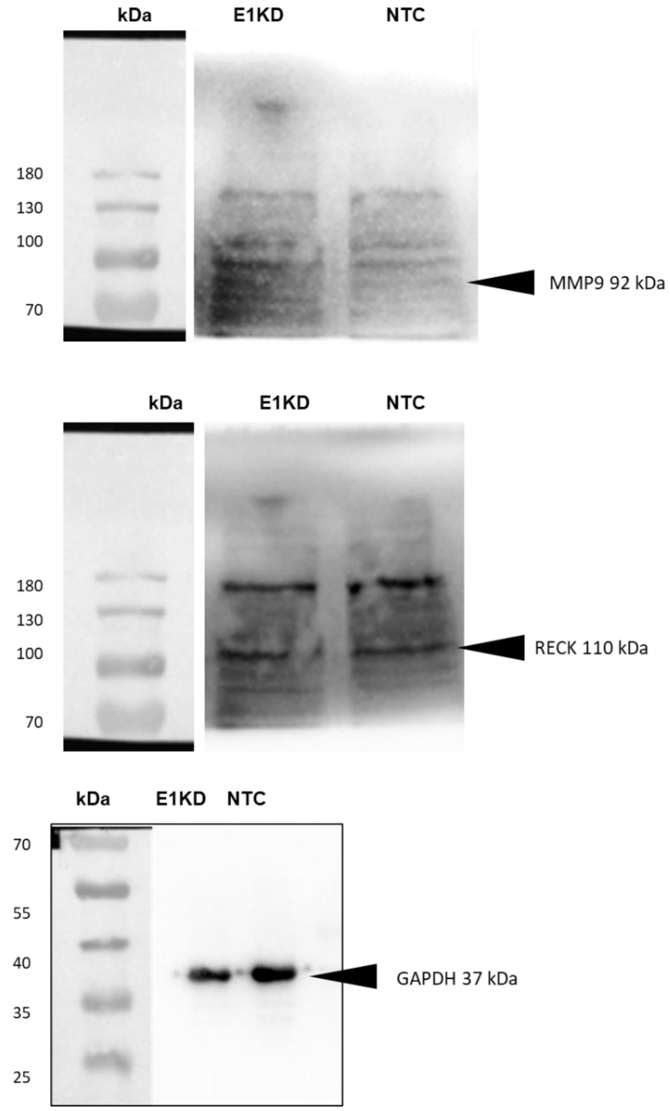

Supplemental Figure 6 (continued). Whole blots from SDS-PAGE and Western blot analysis of MMP9, RECK, and GAPDH. The blots were cut prior to hybridization with antibodies. The ladder and protein band were observed by Chemidoc XRS+ (BIO-RAD, USA); the protein ladder was captured by colorimetric method, whereas the protein band was captured by chemiluminescence method. Three independent experiments were performed as shown in A-C.

References

Baedyananda, F., A. Chaiwongkot, and P. Bhattarakosol. 2017. Elevated HPV16 E1 Expression Is Associated with Cervical Cancer Progression. *Intervirology*. 60:171-180.

Bogovac, Z., M.M. Lunar, B.J. Kocjan, K. Seme, N. Jancar, and M. Poljak. 2011. Prevalence of HPV 16 genomic variant carrying a 63 bp duplicated sequence within the E1 gene in Slovenian women. *Acta Dermatovenerol Alp Pannonica Adriat*. 20:135-139.

Chaiwongkot, A., M. Seki, Y. Suzuki, T. Techawiwat, and P. Bhattarako. 2020. Transcriptional Patterns of High-Risk Human Papillomavirus Types 16, 18, 45, 68b Genes. *Trends in Bioinformatics*. 14:1-12.

Joseph, R., O.P. Srivastava, and R.R. Pfister. 2012. Downregulation of beta-actin gene and human antigen R in human keratoconus. *Investigative ophthalmology & visual science*. 53:4032-4041.

Kannike, K., M. Sepp, C. Zuccato, E. Cattaneo, and T. Timmusk. 2014. Forkhead transcription factor FOXO3a levels are increased in Huntington disease because of overactivated positive autofeedback loop. *J Biol Chem*. 289:32845-32857.

Schröpfer, A., U. Kammerer, M. Kapp, J. Dietl, S. Feix, and J. Anacker. 2010. Expression pattern of matrix metalloproteinases in human gynecological cancer cell lines. *BMC cancer*. 10:553.

Zhou, X.Q., S.Y. Huang, D.S. Zhang, S.Z. Zhang, W.G. Li, Z.W. Chen, and H.W. Wu. 2015. Effects of 5-aza-2'deoxycytidine on RECK gene expression and tumor invasion in salivary adenoid cystic carcinoma. *Brazilian journal of medical and biological research = Revista brasileira de pesquisas medicas e biologicas*. 48:254-260.
